# Becoming Active Bystanders and Advocates: Teaching Medical Students to Respond to Bias in the Clinical Setting

**DOI:** 10.15766/mep_2374-8265.11175

**Published:** 2021-08-19

**Authors:** Michelle York, Kyle Langford, Mario Davidson, Celeste Hemingway, Regina Russell, Maya Neeley, Amy Fleming

**Affiliations:** 1 Medical Student, Vanderbilt University School of Medicine; 2 Assistant Professor, Department of Biostatistics, Vanderbilt University Medical Center; 3 Assistant Professor, Department of Obstetrics and Gynecology, Vanderbilt University Medical Center; 4 Assistant Professor, Department of Medical Education and Administration, Vanderbilt University; 5 Assistant Professor, Department of Pediatrics, Vanderbilt University Medical Center; 6 Associate Dean of Student Affairs, Vanderbilt University School of Medicine

**Keywords:** Microaggression, Bias, Bystander, Anti-Racism, Case-Based Learning

## Abstract

**Introduction:**

Incidents of bias and microaggressions are prevalent in the clinical setting and are disproportionately experienced by racial minorities, women, and medical students. These incidents contribute to burnout. Published efforts to address these incidents are growing, but gaps remain regarding the long-term efficacy of these curricular models. We developed and longitudinally evaluated a workshop that taught medical students a framework to respond to incidents of bias or microaggressions.

**Methods:**

In October 2019, 102 Vanderbilt core clerkship medical students participated in an hour-long, interactive, case-based workshop centered around the 3 D's response behavior framework: (1) direct, (2) distract, and (3) delegate. Participants were surveyed before and after the training, and both qualitative and quantitative data were collected. A refresher workshop was offered 8 months later, which added two additional D's: delay and display discomfort.

**Results:**

After the workshop, respondents’ knowledge of the assessed topics improved significantly, as did their confidence in addressing both personally experienced and witnessed incidents. Respondents initially indicated a high likelihood of using response behaviors to address incidents. The workshop did not consistently modify behavioral responses to experienced or witnessed incidents. Ninety-one percent of respondents agreed the workshop was effective.

**Discussion:**

This workshop provided an effective curriculum to sustainably improve participant knowledge and confidence in responding to incidents of bias and microaggressions. This resource can be adopted by educators at other institutions.

## Educational Objectives

By the end of this activity, learners will be able to:

1. Define the terms bias, microaggression, and active bystander.
2. Summarize the prevalence of bias and microaggressions.
3. Describe the 5 D's model (direct, distract, delegate, delay, and display discomfort) as a framework to respond to incidents of bias and microaggressions as an active bystander.
4. Explain the importance of active bystander advocacy as a model for improving the clinical learning and care environment.
5. Generate possible responses to example cases of bias or microaggressions.

## Introduction

As medicine continues to change at a rapid pace in many facets, one of the most important ways it is changing is the demographic makeup of the physician workforce.^1^ Data published by the AAMC show that over the past 30 years, the demographic makeup of graduating physicians has shifted dramatically, with the number of racial minorities and women increasing significantly.^1^ While the increasing diversity of providers has led to improved quality of patient care,^1^ underrepresented minorities, women, and medical students unfortunately experience substantial bias within the clinical setting.^2–10^ Data collected through the 2020 national AAMC graduation questionnaire showed that 40% of medical school graduates reported experiencing incidents of bias, humiliation, or discrimination based on personal traits at least once during training.^11^ These negative experiences are commonplace at every step in the training path and limit the culture of inclusivity while contributing to burnout.^12^

Despite extensive literature supporting important cultural and systemic issues in medicine, the integration of microaggressions training and anti-racism training into medical school curricula is a relatively new effort.^13–17^ As part of the Anti-racism in Medicine Collection, *MedEdPORTAL* has recently published multiple approaches aimed at teaching supervisors or medical students how to respond to microaggressions.^15–18^ These excellent workshops all adopt slightly different approaches, and each reports success in improving respondents’ confidence in identifying and responding to microaggressions immediately after the intervention. Introducing multiple approaches adds necessary depth to the literature and offers programs the opportunity to identify the best fit for their curriculum and student body. Our curriculum adds a unique contribution to this collection through our behavioral response framework.

Bystander training has recently gained popularity on college and high school campuses to target power-based mistreatment, particularly through the Green Dot program. The Green Dot program was originally developed to target sexual violence on college campuses, and studies have demonstrated its efficacy as a model for reducing interpersonal violence.^19–21^ With clear evidence showing that discrimination via biases and microaggressions is a prevalent issue in the clinical workspace, in conjunction with evidence supporting the success of bystander intervention training, we developed, taught, and evaluated an iterative student-led workshop aimed at training clinical medical students to be active bystanders. This workshop provided participants with a framework of various behavioral response types that could be applied to different kinds of incidents of witnessed or experienced bias and microaggressions. The behavioral response framework consists of the 5 D's or response types, which include direct, distract, delegate, delay, and display discomfort.

## Methods

This educational curriculum received exempt status from the Vanderbilt University Institutional Review Board.

### Curriculum Context

At the Vanderbilt University School of Medicine, our medical school program features a condensed, 1-year, preclinical curriculum that allows students to participate in the core clinical clerkships during the second year of training. Given that the core clerkships are usually a student's first longitudinal immersion in the clinical environment and that we sought to teach clinical medical students, we implemented the workshop after clerkship students completed their first core rotation. This allowed students to (1) better contextualize the topic of the workshop, as they may have witnessed or experienced bias or microaggressions during their first rotation in the hospital, and (2) apply the skills soon after the workshop. In October 2019, 102 Vanderbilt core clerkship students completed the first bystander training workshop. Of note, we offered participants a refresher workshop online via Zoom at 8 months after the initial training session. The refresher workshops were performed in groups of 10–20 students and included two additional D's: delay and display discomfort. The dean of student affairs (who holds a master's degree in health professions education) and a third-year medical student coled the workshops. The faculty facilitator had extensive experience in medical student education and workshop facilitation. The student facilitator had no formal prior experience in medical education or teaching. In preparation for the workshop facilitation, the student developed a presentation script and received coaching around teaching and facilitation skills for leading a workshop from the faculty cofacilitator.

### Implementation and Logistics

We designed a bystander training educational workshop centered around providing a behavioral response framework to participants. As an overview, this workshop included a brief didactic review of the important terminology and relevant background data, instruction on the behavioral response framework, and a moderated, interactive review of three to four example cases of bias in the workplace. Initially, we used the Green Dot 3 D's; however, after an insightful lecture from a visiting professor from the Emory University School of Medicine, Dr. Kimberly Manning, we expanded our training with her permission to incorporate two additional D's from her 5 D's Upstander Training. The purpose of the 5 D's, which are listed below, is to offer a variety of possible responses so that the framework is applicable to many settings.

1. Direct: Verbally address the incident and respond to the perpetrator in the moment.
2. Distract: Defuse the situation by shifting the attention or focus of the perpetrator to prevent further harm.
3. Delegate: Entrust the response to another individual who may be able to better approach the individual and engage them in addressing the incident.
4. Delay: Discuss the situation with the perpetrator or victim at a later time and/or different setting.
5. Display Discomfort: Express nonverbal discomfort or concern in response to the incident.

We taught medical students the 5 D's framework using an interactive workshop that allowed them time to practice applying this framework to example microaggressions. The presentation slides, the facilitator guide, and the behavioral response framework description are included in Appendices A–C, respectively. Facilitators taught the 50-minute workshop during the students’ lunch hour. No presession preparation was required for participants. Students sat at tables in groups of six to eight.

We began the workshop with a short introduction, during which we introduced our speakers, reviewed the time line and objectives for the session, and allowed participants to complete a preworkshop survey. We used this time to acknowledge that not everyone experiences biases and microaggressions in the same way and that although some students may experience these incidents at a much higher frequency than others, it is a shared responsibility to foster a community of safety for all students, staff, and patients. We explained that all of us are prone to commit microaggressions and discussed the importance of learning and seeking educational resources. We also established expectations and guidelines for group interactions to facilitate sharing diverse perspectives and learning together.

Workshop leaders then began a brief, interactive lecture. This started with a review of important terminology, during which the facilitators engaged the group members on their understanding before providing shared definitions for the following terms: bias, microaggression, and active bystander. We emphasized that, though the term may suggest otherwise, the impact of microaggressions can be significantly distressing for the targeted individual or victim. Facilitators also polled participants on their past experiences with incidents of bias and microaggressions in the clinical setting using Poll Everywhere. Facilitators provided context for the workshop by reviewing background data on the prevalence and impacts of bias and microaggressions.

We then reviewed the response behavior framework. Facilitators took the first few minutes to explain the foundation of the framework by discussing the Green Dot program and its efficacy at reducing power-based mistreatment. We then walked through each D, providing definitions (as above) and examples of each. We explained to participants that there was no single superior response type and that the response type selected would vary depending on variables pertaining to the scenario, such as the individual's relationship to the microaggressor, witnessed versus experienced incidents, sense of safety, and setting. We emphasized that taking any form of action was a meaningful and important step in contributing to a safe patient care and clinical learning environment.

The next portion of the workshop involved application of the D's framework using two to three cases. The example cases used in our workshops are provided in Appendix D. Of note, because we offered the workshop in two unique settings (in person and virtually), we trialed two different methods—role-play and response generation—for structuring discussion of these cases.

#### Role-play

This model was trialed in an in-person setting. We divided participants into groups of three. We provided instructions for using role-play within the small groups. After reading the case together, groups discussed which D they would realistically select. Then, participants used role-play to practice the direct response method, as this is typically most challenging. The roles for each case included the perpetrator, the responder, and the observer of the situation. We instructed participants to rotate to a new role for each case. After a few minutes of practice, facilitators asked participants to return to the large group for discussion and reflection on the case. We repeated this model for the additional selected cases.

#### Response generation

We piloted this model over Zoom during the optional refresher workshop. In this setting, students had attended the original workshop. After briefly reviewing the 5 D's framework with participants and providing examples of each response type, we provided participants with a sample case that was read aloud to the large group. Participants were divided into breakout groups of three to four. In the breakout rooms, participants discussed which D they were most likely to use and generated possible ways to respond to the case (focusing on direct, distract, and delay methods). Facilitators then asked participants to return to the large group for a debrief reviewing possible responses and perceived barriers to intervening. Notes were taken by facilitators during this time. We repeated this process for the additional cases. After completion of the virtual refresher workshop, we sent out a summarized list of generated responses to the cohort of participants for their reference.

Finally, facilitators concluded the workshop with a short debrief session. During this time, we asked for general reflections from the group and requested that participants share one area or D they could commit to trying in the weeks following the session. We also used this time to distribute a postworkshop survey (Appendix E).

The student facilitator led a 1-hour focus group with four student participants. A focus group facilitator guide is included in Appendix F. Faculty and student leaders developed discussion questions for the session, which aimed to understand what elements of the workshop were effective and where to focus improvement efforts. Workshop participants indicated their interest in joining the focus group on the survey collected immediately postworkshop. With permission from the participants, we recorded the audio of the focus group discussion in an anonymous fashion. Any participant identifiers were removed from the transcript prior to analysis.

### Evaluation

To evaluate the workshop and its impacts on participants’ knowledge, confidence, and behavioral responses, we collected both qualitative and quantitative data from participants via surveys collected immediately pre- and postworkshop, as well as at 1 month, 3 months, and 8 months postworkshop. Of note, the display discomfort response was assessed only at the 8-month postworkshop survey.

We collected quantitative data using survey questions that participants rated based on Likert scales (e.g., 1 = *strongly agree,* 5 = *strongly disagree*; Appendix E). We compared the results of the matched questions from the pre- and postworkshop surveys using two-sample paired *t* tests with unequal variances. We analyzed quantitative data using Microsoft Excel. To look at long-term efficacy, we also conducted this analysis comparing preworkshop and 8-month postworkshop data.

We analyzed qualitative data gathered from free-response survey questions and the 1-hour focus group session using principles of grounded theory. Two independent coders identified emergent and a priori themes. NVivo (QSR International) was used by coders. Intercoder reliability was found to be greater than 92% across the analyzed documents. Coders reviewed the data and reconciled the minor coding discrepancies.

## Results

A total of 102 students participated in the initial workshop. Respondent demographics are described in Table 1. Although the presurvey achieved high response rates with regard to the listed demographic identifiers, we did not perform subgroup analyses on follow-up surveys due to low response rates (Table 2).

**Table 1.**
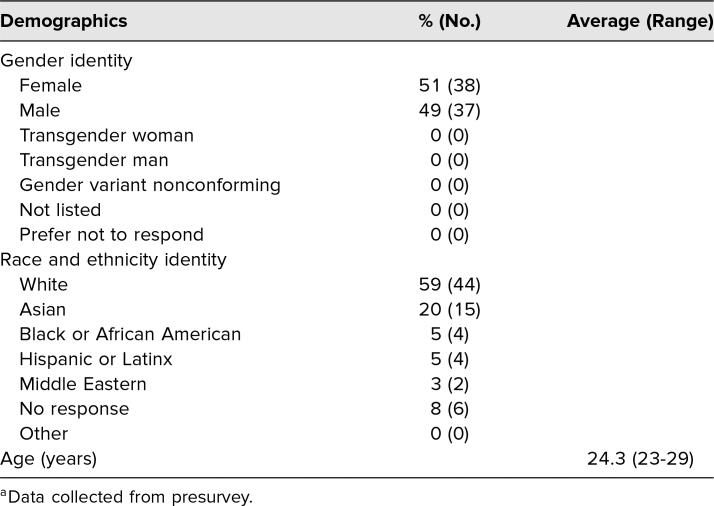
Respondent Demographics^a^

**Table 2.**
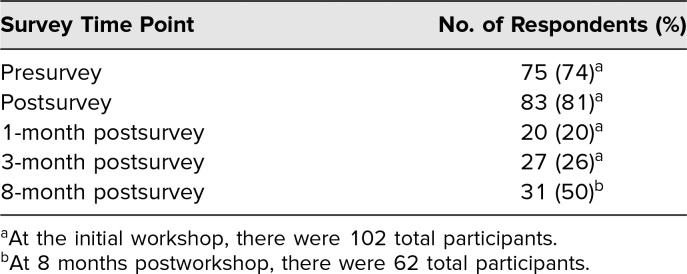
Survey Response Rates

Respondents’ confidence in addressing both personally experienced and witnessed incidents of bias and microaggressions improved significantly both immediately after the workshop (2.36 vs. 2.99, *p* < .05) and at 8 months postworkshop (2.36 vs. 3.07, *p* < .05; Table 3). Additionally, compared to preworkshop data, reported knowledge of all assessed topics improved significantly (*p* < .05) immediately after completion of the workshop and at 8 months postworkshop (Table 4).

**Table 3.**
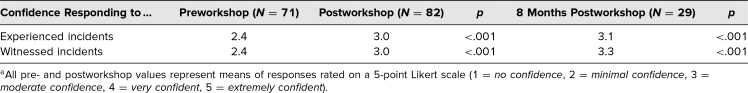
Reported Confidence in Responding to Experienced and Witnessed Incidents at Pre- and Postworkshop Time Points^a^

**Table 4.**
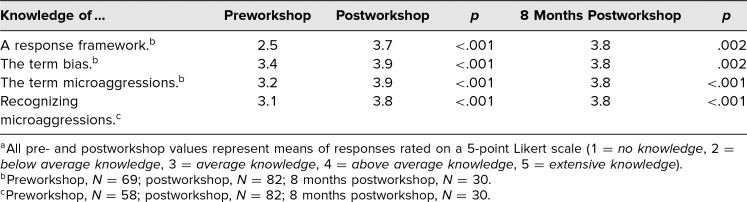
Comparison of Reported Knowledge at Pre- and Postworkshop Time Points^a^

Immediately following completion of the workshop, respondents indicated a high likelihood of using each of the assessed response behaviors (direct, distract, delay, and delegate) to address both experienced and witnessed incidents. The use of direct, distract, delay, and delegate responses did not change significantly after the workshop with regard to experienced or witnessed incidents. Assessed only at the 8-month time point, 100% of respondents reported that they had used the display discomfort behavior “sometimes” or “often” in response to both witnessed and experienced incidents.

Common reported barriers to responding to incidents included (1) concern regarding the clinical hierarchy, (2) questioning whether the incident was important enough to address, (3) uncertainty regarding the individual's interpretation of the event, and (4), specifically with witnessed incidents, uncertainty regarding the victim's preferences for intervention or assistance.

Respondents indicated a high degree of satisfaction with the workshop, with 91% agreeing that the initial workshop was effective and 96% of 8-month postsurvey respondents agreeing that the virtual refresher workshop was effective. Still, after completion of the refresher workshop, 61% of respondents desired additional training, while 19% reported that the two workshops were enough.

Based on qualitative analysis, the most effective aspects of the training were cases (37% of respondents), response generation (32% of respondents), and the response behavior framework (22% of respondents). Two students reported their views on the most effective components:

• “They were realistic cases that people could take in different directions based on experiences that they had that were very similar.”
• “[Discussion of response behaviors was] helpful because it helped provide framework for what we could actually say.”

Furthermore, all four focus group participants agreed that this was a needed curricular change. One student spoke to this sentiment: “I thought it was that needed activity. Obviously, any workplace, but especially in the hospital, there's always things that come up that you want to address and feel like you avoid instead of addressing it. So, I'm glad we had that kind of a session.”

Finally, the most common suggested areas for improvement identified within the qualitative data included reducing time spent on definitions and using participant-generated case examples.

## Discussion

Given the high prevalence of bias and microaggressions in the clinical setting and the known negative cumulative impacts that may limit or slow necessary efforts toward improving diverse representation in medicine, targeting these incidents is an important area of investigation. Our findings suggest that this resource provided a unique and efficient training model consisting of one to two workshops that significantly and sustainably improved respondents’ confidence in addressing witnessed or experienced incidents of bias or microaggressions, as well as their knowledge of the assessed topics.

Our curriculum contributes a unique perspective to the literature surrounding microaggressions workshops via a few key attributes. First, the 5 D's model offers a dynamic and adaptable response framework that can be applied in a meaningful way in many scenarios, including interdisciplinary and patient care settings. While many frameworks use the direct, distract, and delegate responses, the two additional D's contributed by Dr. Kimberly Manning—delay and display discomfort—are important additions to consider and are not found in other published curricula. The intentional inclusion of a delayed approach may allow individuals necessary time to reflect on the scenario or decompress with a mentor or friend, or it may allow a responder the opportunity to respond in a more private or comfortable setting. Meanwhile, the display discomfort approach provided a nonverbal cue that can be an effective immediate form of feedback to the perpetrator.

Our educational innovation also adds to the literature in that we studied our respondents over a longitudinal period and studied participants’ self-reported behavioral responses. As our data collection spans over 8 months following the workshop, our curricular analysis offers insight into the longitudinal efficacy of these types of workshops, which is important when considering future efforts. While our results suggested that respondent knowledge and confidence in responding improved after the workshop and that this improvement was sustained over time, our data were less conclusive when looking at behavioral impacts. Our results suggested that immediately following the workshop, respondents were likely to use the response behaviors they had learned, but based on longitudinal follow-up, we saw that predicted behavioral changes were not consistently achieved. It was difficult to confidently draw conclusions on this intervention's behavioral impacts, as we saw low reported response rates on the 1-month, 3-month, and 8-month postsurveys. Thus, while initial data suggest that this intervention may not achieve significant behavioral changes with respect to direct, distract, delay, and delegate methods, further exploration is needed. Importantly, respondents indicated a high rate of using the display discomfort approach, which was assessed only at the 8-month time point. Longitudinal analysis of this response behavior is needed to assess for further conclusions.

Finally, much of the existing literature on this topic includes workshops that have been designed for specific participant populations. While the data shown here concern the impact on clinical medical students, this curriculum (with minor adaptations to the example cases) has successfully been given to preclinical and clinical medical students, residents, fellows, speech-language pathology students, audiology students, and nursing students, demonstrating its adaptability.

Since introducing this workshop to the clerkship medical students, we have received institutional support to provide it to the remaining classes within the School of Medicine, as well as to a group of chief residents and fellows within our institution. Through these experiences, we have learned that this model and workshop are extremely adaptable to many different trainee backgrounds. As workshop facilitators, with each new audience we modified the time spent on and content of the background information, as well as the case examples. For example, when working with our first-year medical students, we dedicated extra time to providing context and examples of incidents given the students' limited clinical exposure. When working with the chief residents and fellows, we shortened and tailored the background information to be more applicable to their position in the training pathway. We also chose to modify the case examples used with residents and fellows to include themes of intervening on behalf of a student or patient. It is important and useful to note that the 5 D's response model was kept constant with each audience, which provided community members with a shared language for discussion and intervention.

Additionally, it is important for future educators to know that while our results indicated that this workshop was very well received, some respondents remained resistant to the personal relevance of the discussed concepts. A strategy that may help facilitators professionally and constructively address this reaction is to acknowledge that every audience member brings unique and valuable perspectives based upon their past experiences and to offer the reminder that this model is applicable for all participants regardless of how frequently they experience these incidents as it provides actionable lessons for observers as well.

This curriculum has many strengths. First, we evaluated and analyzed multiple variables, including perceived efficacy, knowledge, confidence, and behavioral responses. This multifactorial assessment allowed us to better understand the extent of our workshop's impact. Additionally, our longitudinal data collection demonstrated that the workshop's effects persist over time.

There were several limitations to acknowledge in this educational innovation. First, the uniqueness of the Vanderbilt School of Medicine clinical curriculum and the low response rates on longitudinal follow-up surveys may limit the generalizability of our results. Furthermore, as the educators were known to respondents, this may have been a source of bias (either positive or negative) in our results. Additionally, there was potential for the Hawthorne effect to bias the results, as respondents knew they were being assessed on the described aims, including their behaviors. Finally, our survey methodology relied on participants’ self-assessment and did not include practical skills-based evaluations, which limited the strength of our conclusions with respect to our educational objectives.

Given our results and current limitations, there are many exciting improvements and new directions to pursue. With regard to the workshop elements, incorporating cases submitted or shared by participants may further improve participant satisfaction and perceived utility. This personalized aspect may also improve engagement from individuals who less frequently experience these incidents. Additionally, it is worthwhile to explore which barriers to responding are modifiable. While the clinical hierarchy is more difficult to modify, offering this training to residents, fellows, and faculty is a crucial step to improving cultural awareness, decreasing incident prevalence (as many students experience incidents from residents or attendings^4–9,18^), and improving social safety by flattening the social hierarchy. Also, given that uncertainty regarding victim preferences is a common reason for deferring a response for witnessed incidents, this may be an important area to explore and incorporate into the teaching. Furthermore, as many students expressed a desire for further training, incorporating additional workshops or teaching methods into our curriculum may improve and reinforce an individual's confidence in interpreting the event and its importance. These methods may include skill-building sessions or assessments in standardized patient settings.

From a broader perspective, distributing and implementing this workshop at other institutions and within other educational disciplines (e.g., science, technology, engineering, and mathematics fields) will allow for improved understanding of this model's efficacy and validity.

After successful implementation of this educational curriculum at our institution, the described model and teaching guides can serve as useful resources to other institutions and educators that are committed to anti-racism training through improving environmental safety and cultural inclusivity within medicine.

## Appendices


Bystander Training.pptxFacilitator Guide.docxResponse Framework Handout.docxExample Cases.docxSurveys.docxFocus Group Facilitator Guide.docx
*All appendices are peer reviewed as integral parts of the Original Publication.*
