# Supplementary material for: Becoming Active Bystanders and Advocates: Teaching Medical Students to Respond to Bias in the Clinical Setting
Source: MedEdPORTAL. 2021 Aug 19;17:11175. doi: 10.15766/mep_2374-8265.11175 (PMC8374028; doi:10.15766/mep_2374-8265.11175)
Supplement: Supplementary file 1 — Bystander Training.pptxFacilitator Guide.docxResponse Framework Handout.docxExample Cases.docxSurveys.docxFocus Group Facilitator Guide.docx [file mep_2374-8265.11175-s001.zip › A. Bystander Training.pptx]

## Slide 1
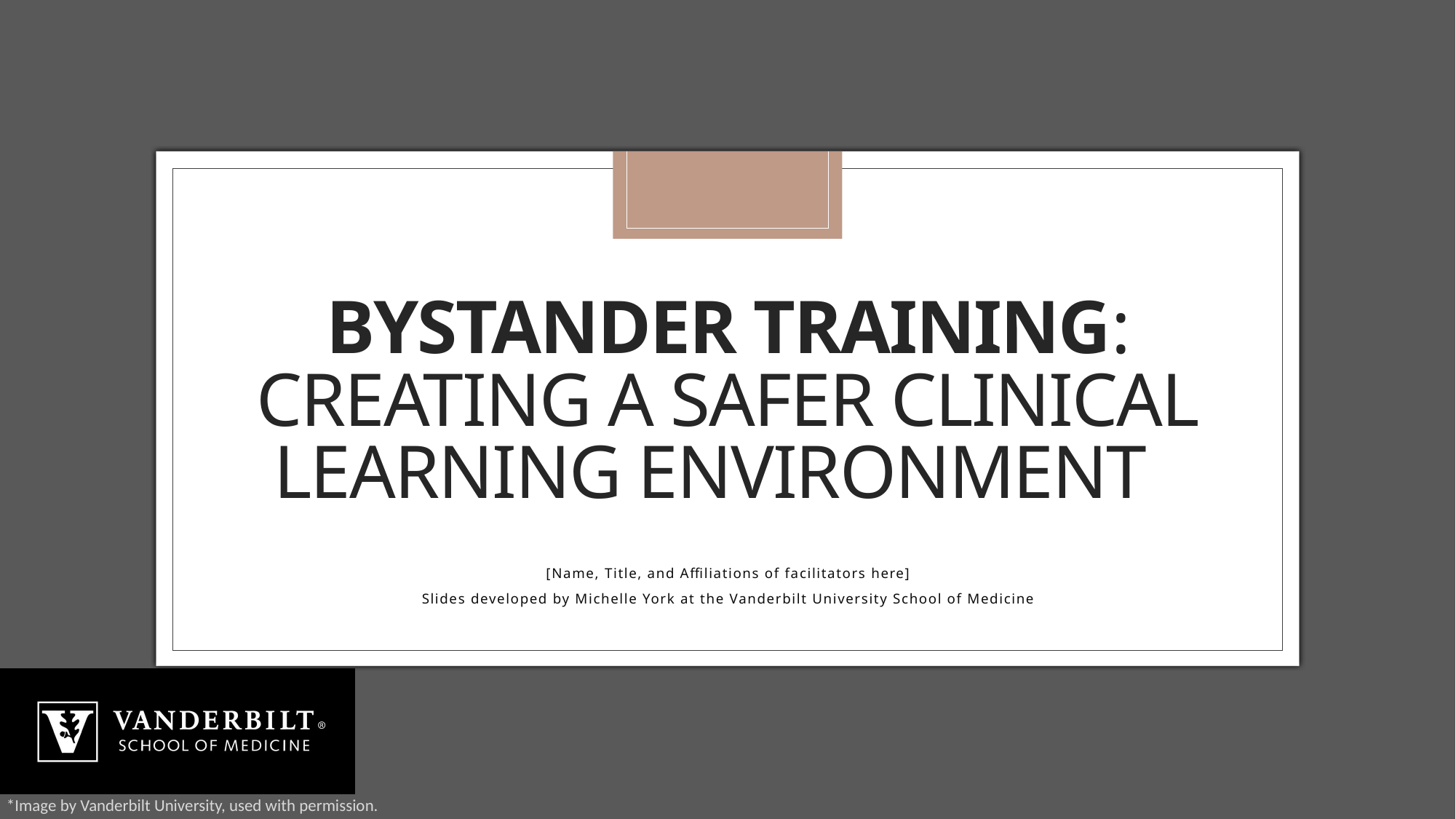

# Bystander Training: Creating a safeR Clinical learning environment
[Name, Title, and Affiliations of facilitators here]
Slides developed by Michelle York at the Vanderbilt University School of Medicine
*Image by Vanderbilt University, used with permission.

## Slide 2
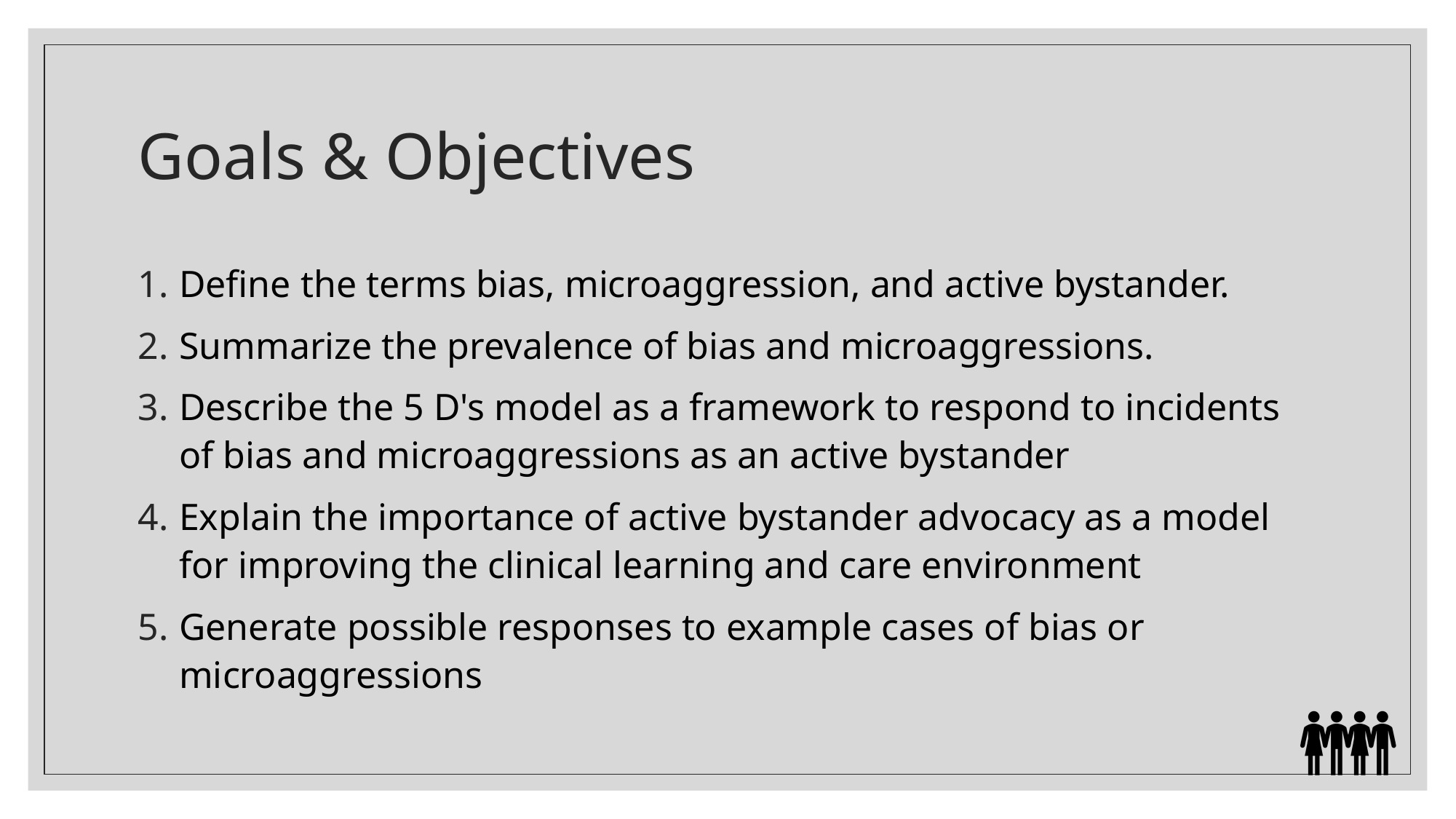

# Goals & Objectives
Define the terms bias, microaggression, and active bystander.
Summarize the prevalence of bias and microaggressions.
Describe the 5 D's model as a framework to respond to incidents of bias and microaggressions as an active bystander
Explain the importance of active bystander advocacy as a model for improving the clinical learning and care environment
Generate possible responses to example cases of bias or microaggressions

## Slide 3
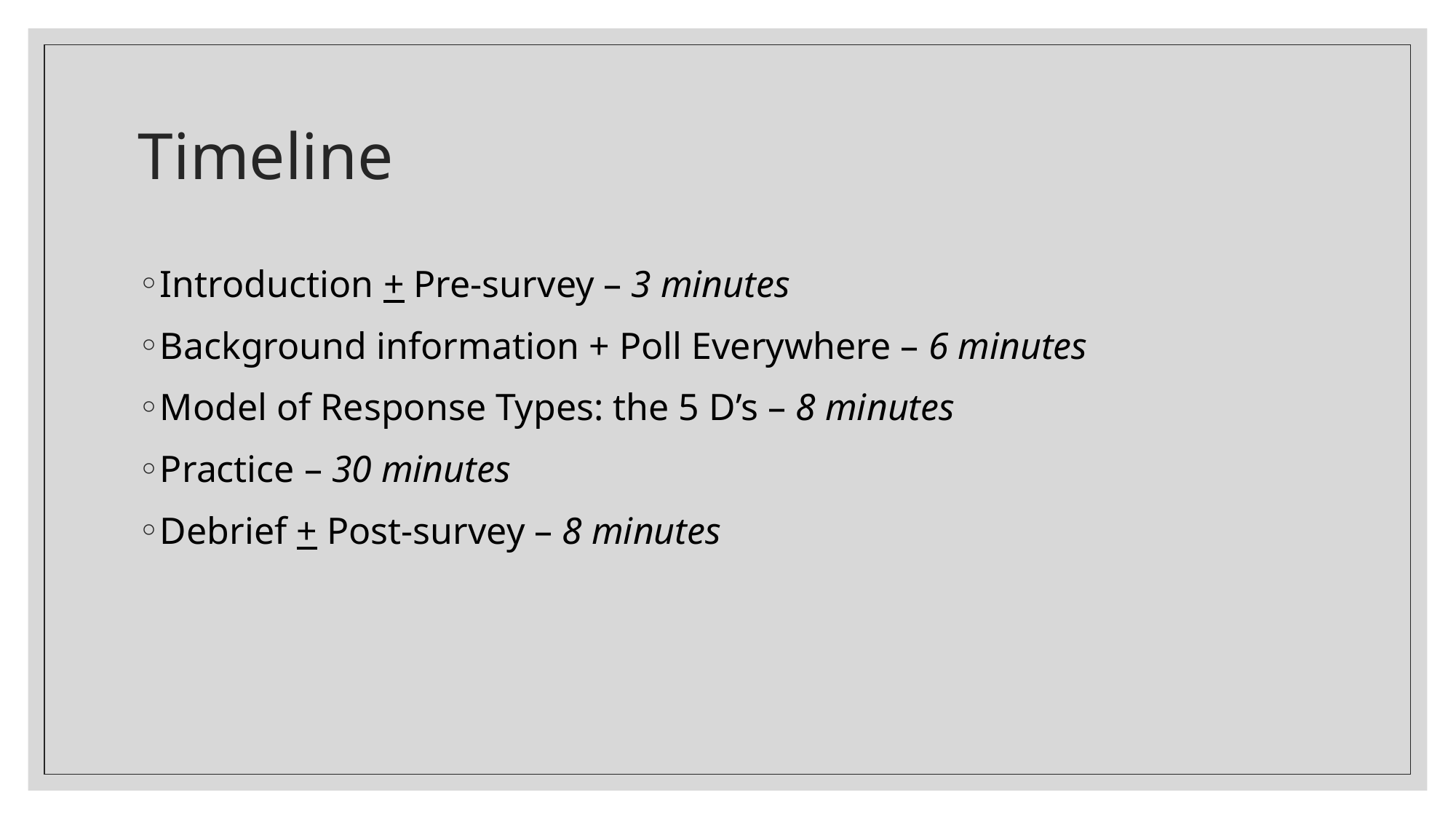

# Timeline
Introduction + Pre-survey – 3 minutes
Background information + Poll Everywhere – 6 minutes
Model of Response Types: the 5 D’s – 8 minutes
Practice – 30 minutes
Debrief + Post-survey – 8 minutes

## Slide 4
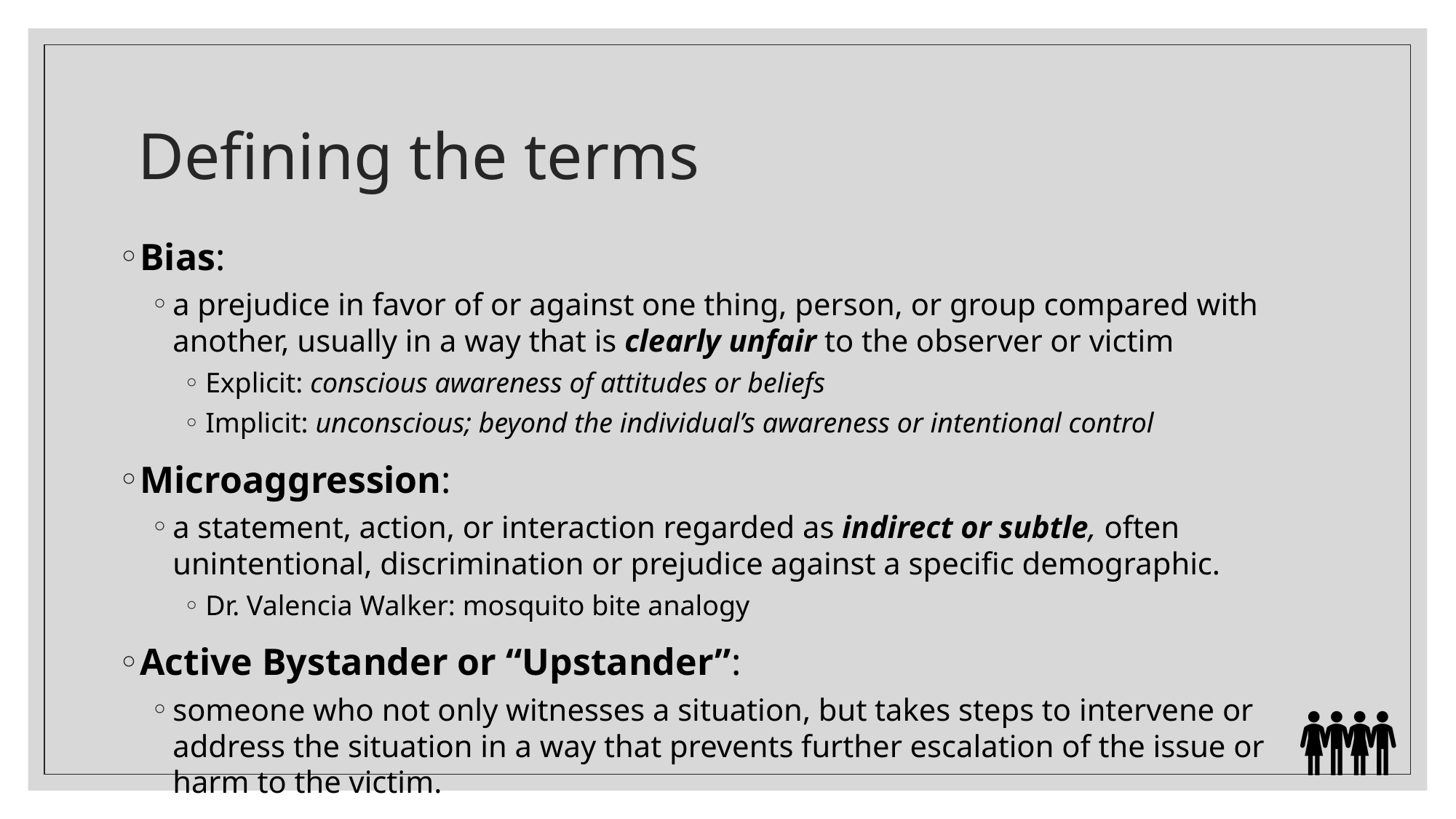

# Defining the terms
Bias:
a prejudice in favor of or against one thing, person, or group compared with another, usually in a way that is clearly unfair to the observer or victim
Explicit: conscious awareness of attitudes or beliefs
Implicit: unconscious; beyond the individual’s awareness or intentional control
Microaggression:
a statement, action, or interaction regarded as indirect or subtle, often unintentional, discrimination or prejudice against a specific demographic.
Dr. Valencia Walker: mosquito bite analogy
Active Bystander or “Upstander”:
someone who not only witnesses a situation, but takes steps to intervene or address the situation in a way that prevents further escalation of the issue or harm to the victim.

## Slide 5
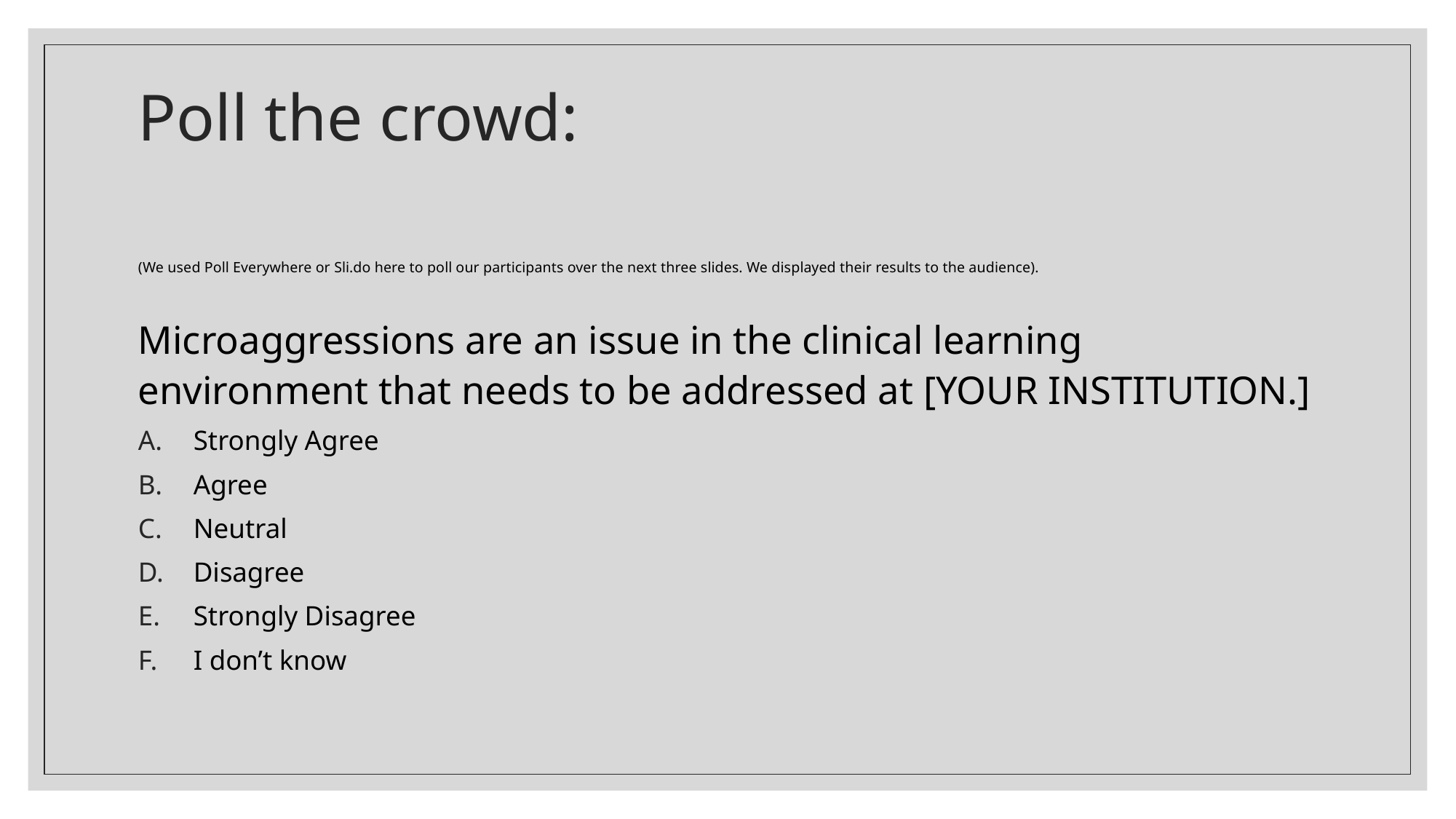

# Poll the crowd:
(We used Poll Everywhere or Sli.do here to poll our participants over the next three slides. We displayed their results to the audience).
Microaggressions are an issue in the clinical learning environment that needs to be addressed at [YOUR INSTITUTION.]
Strongly Agree
Agree
Neutral
Disagree
Strongly Disagree
I don’t know

## Slide 6
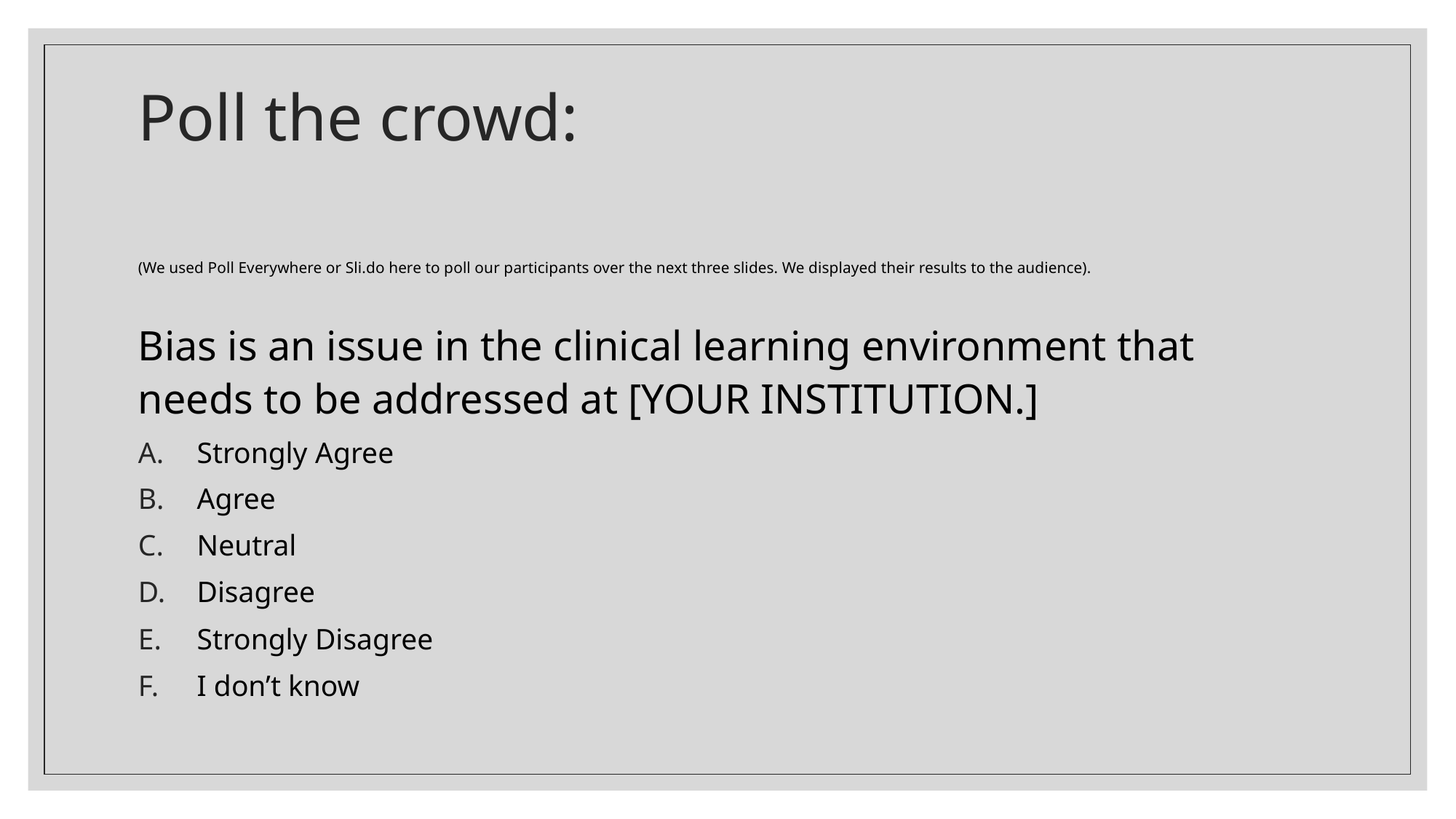

# Poll the crowd:
(We used Poll Everywhere or Sli.do here to poll our participants over the next three slides. We displayed their results to the audience).
Bias is an issue in the clinical learning environment that needs to be addressed at [YOUR INSTITUTION.]
Strongly Agree
Agree
Neutral
Disagree
Strongly Disagree
I don’t know

## Slide 7
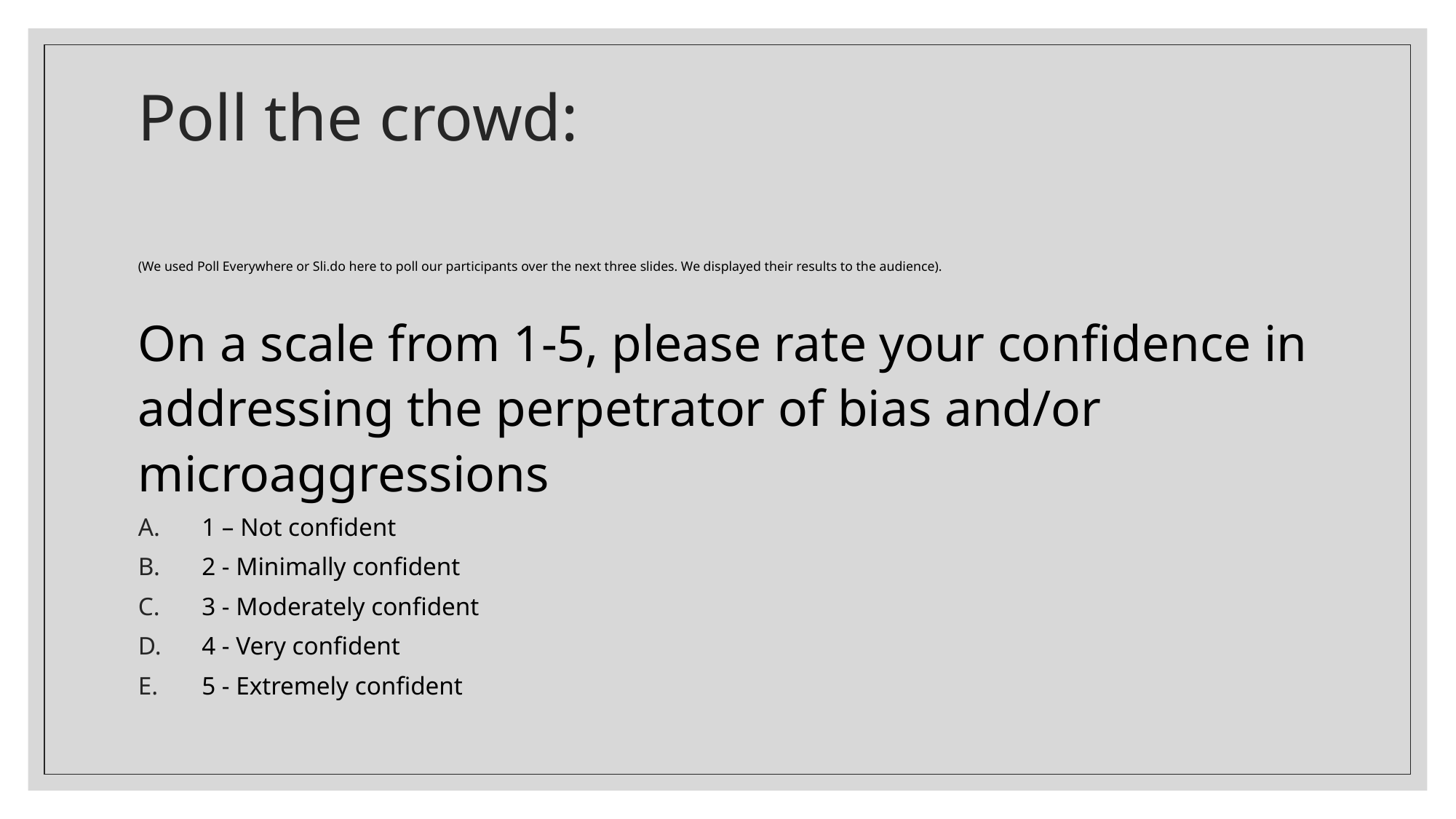

# Poll the crowd:
(We used Poll Everywhere or Sli.do here to poll our participants over the next three slides. We displayed their results to the audience).
On a scale from 1-5, please rate your confidence in addressing the perpetrator of bias and/or microaggressions
1 – Not confident
2 - Minimally confident
3 - Moderately confident
4 - Very confident
5 - Extremely confident

## Slide 8
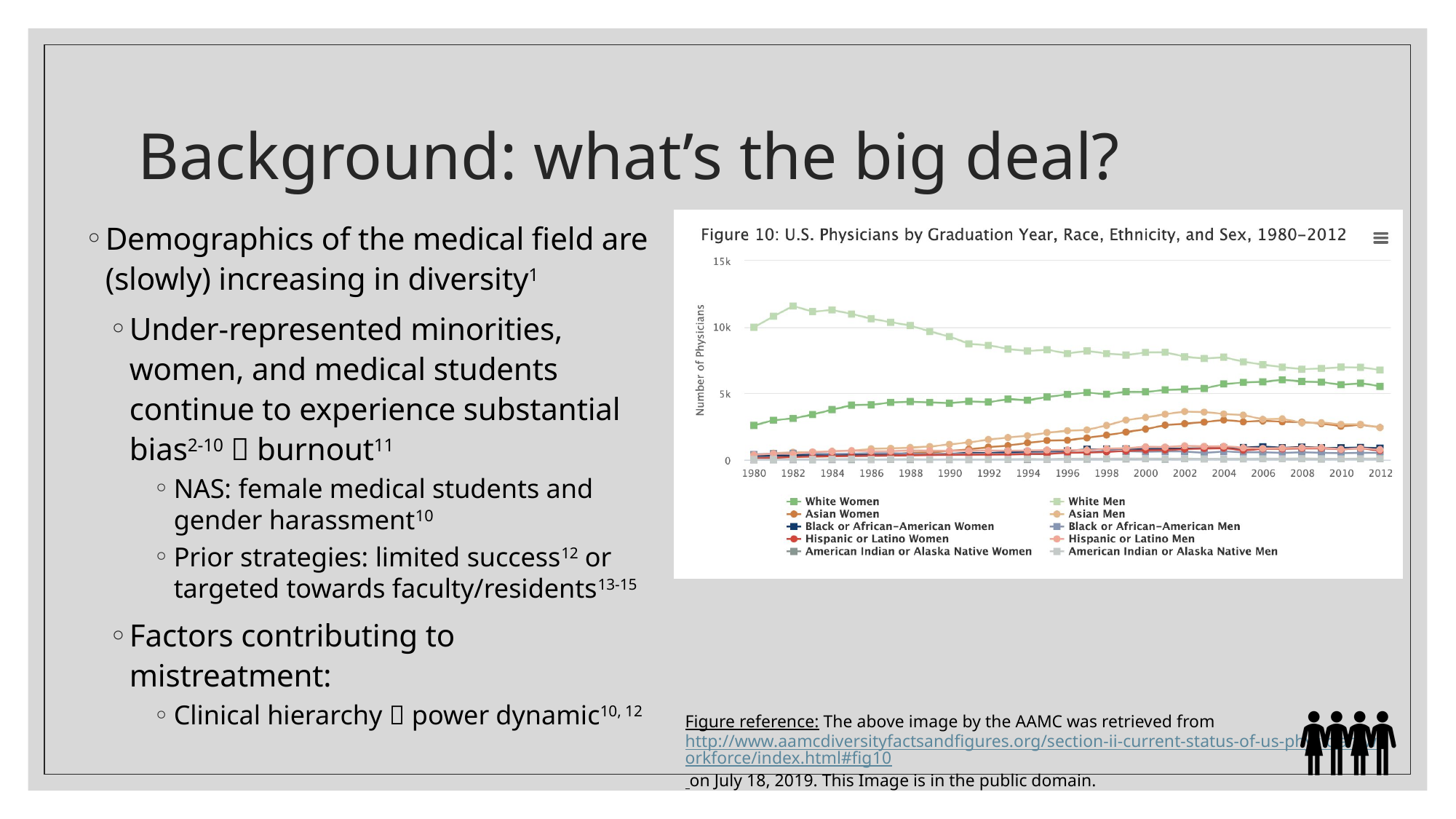

# Background: what’s the big deal?
Demographics of the medical field are (slowly) increasing in diversity1
Under-represented minorities, women, and medical students continue to experience substantial bias2-10  burnout11
NAS: female medical students and gender harassment10
Prior strategies: limited success12 or targeted towards faculty/residents13-15
Factors contributing to mistreatment:
Clinical hierarchy  power dynamic10, 12
Figure reference: The above image by the AAMC was retrieved from http://www.aamcdiversityfactsandfigures.org/section-ii-current-status-of-us-physician-workforce/index.html#fig10 on July 18, 2019. This Image is in the public domain.

## Slide 9
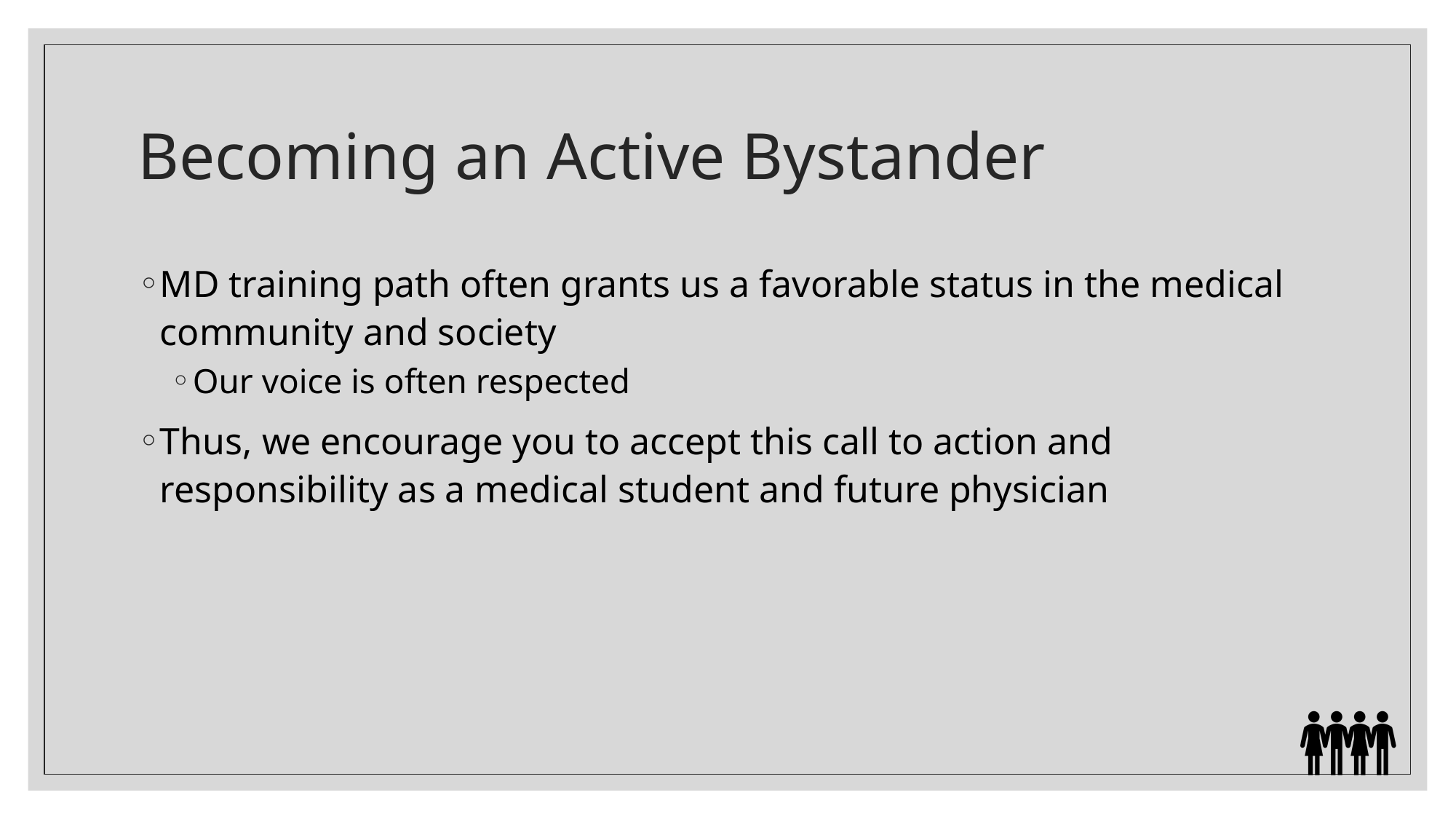

# Becoming an Active Bystander
MD training path often grants us a favorable status in the medical community and society
Our voice is often respected
Thus, we encourage you to accept this call to action and responsibility as a medical student and future physician

## Slide 10
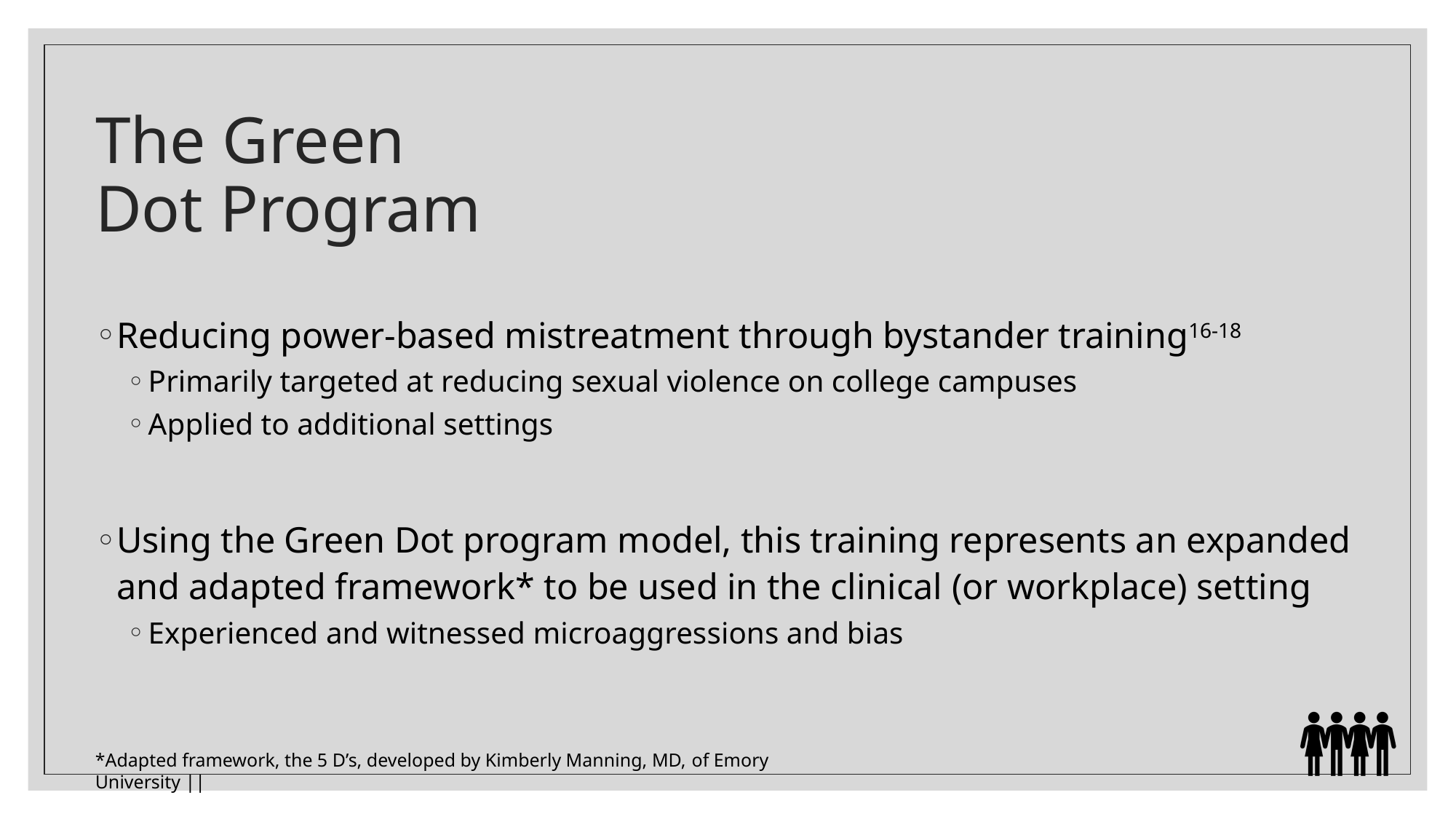

# The Green Dot Program
Reducing power-based mistreatment through bystander training16-18
Primarily targeted at reducing sexual violence on college campuses
Applied to additional settings
Using the Green Dot program model, this training represents an expanded and adapted framework* to be used in the clinical (or workplace) setting
Experienced and witnessed microaggressions and bias
*Adapted framework, the 5 D’s, developed by Kimberly Manning, MD, of Emory University ||

## Slide 11
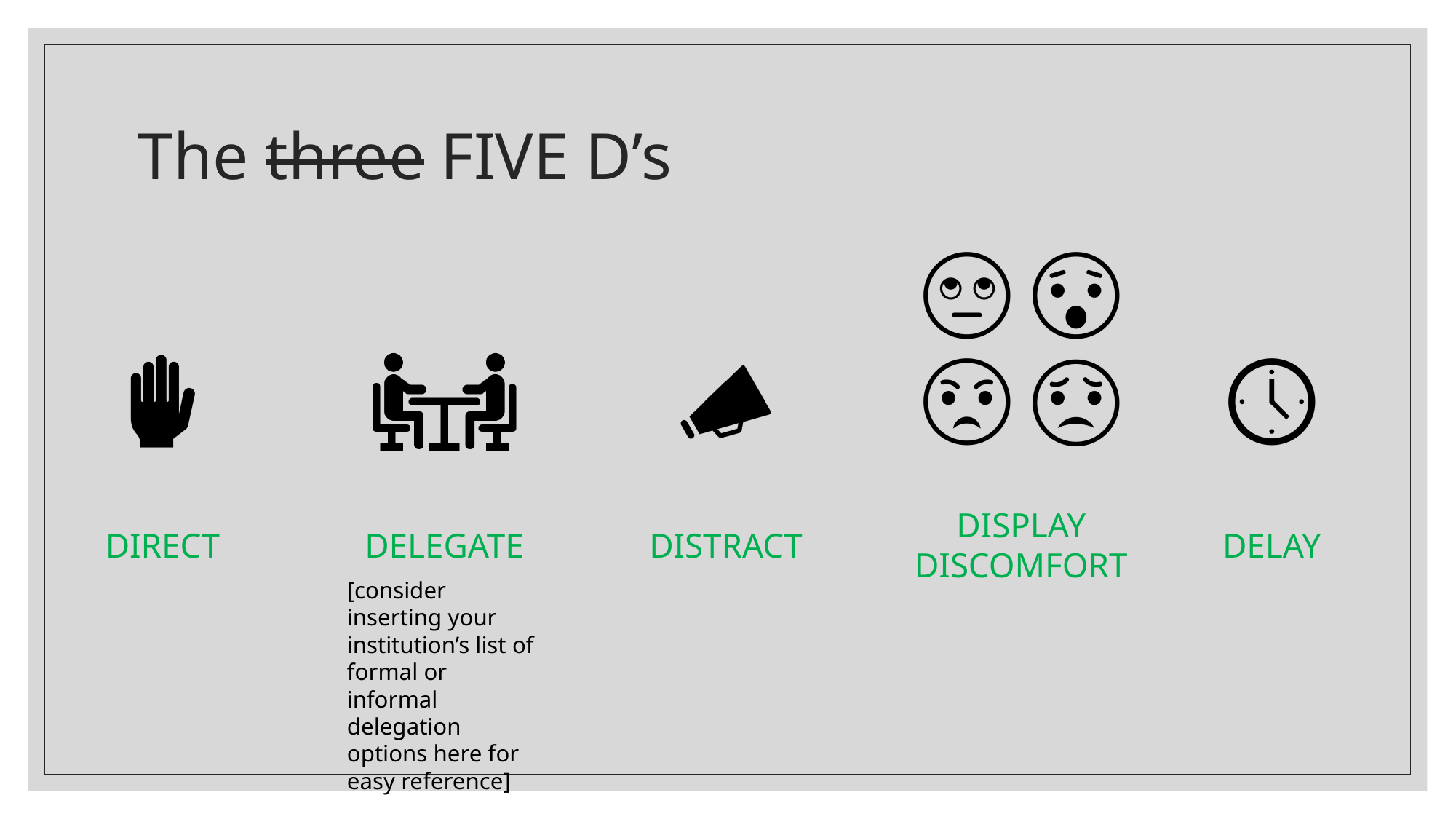

# The three FIVE D’s
DISPLAY
DISCOMFORT
DIRECT
DISTRACT
DELAY
DELEGATE
[consider inserting your institution’s list of formal or informal delegation options here for easy reference]

## Slide 12
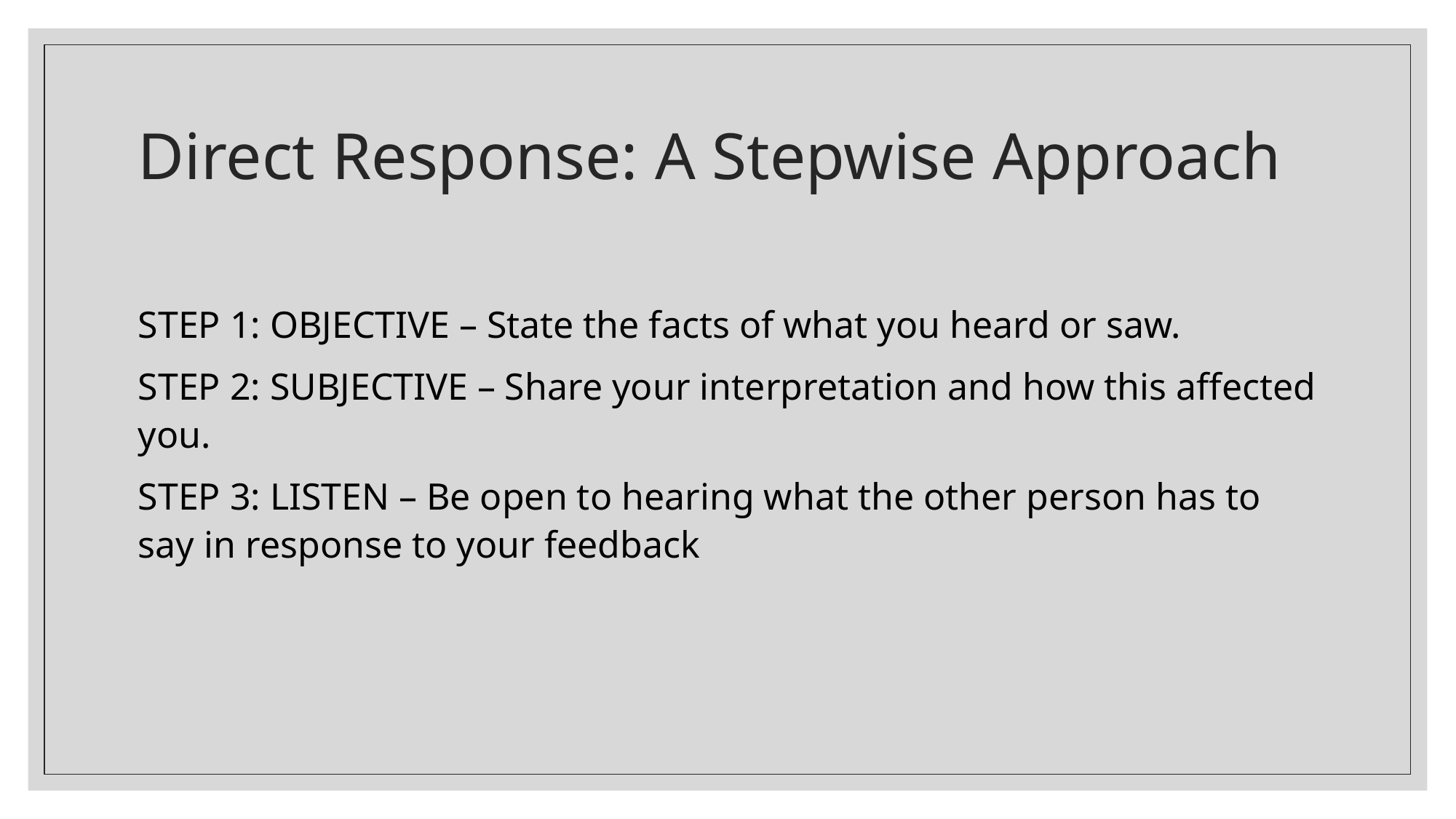

# Direct Response: A Stepwise Approach
STEP 1: OBJECTIVE – State the facts of what you heard or saw.
STEP 2: SUBJECTIVE – Share your interpretation and how this affected you.
STEP 3: LISTEN – Be open to hearing what the other person has to say in response to your feedback

## Slide 13
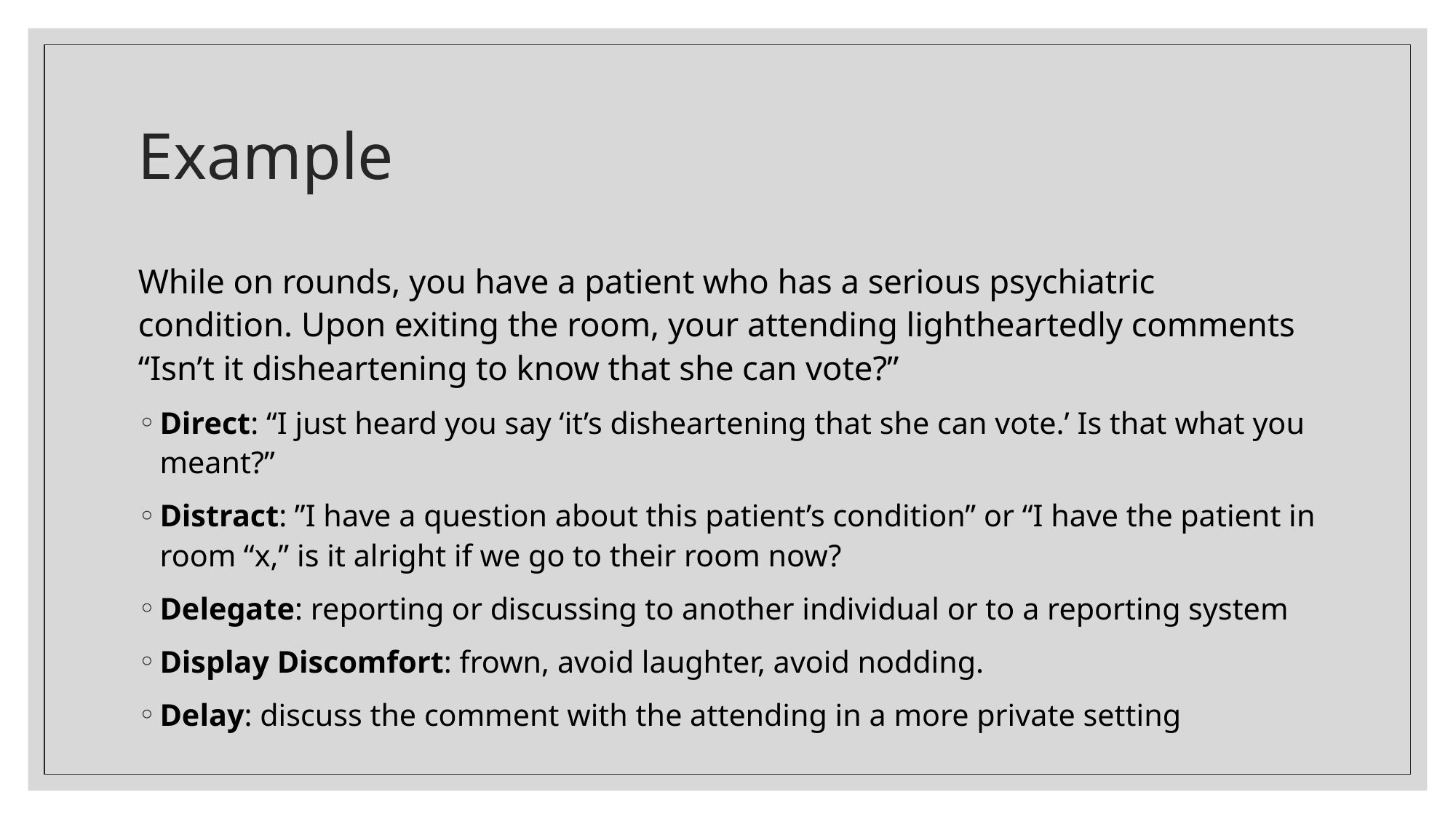

# Example
While on rounds, you have a patient who has a serious psychiatric condition. Upon exiting the room, your attending lightheartedly comments “Isn’t it disheartening to know that she can vote?”
Direct: “I just heard you say ‘it’s disheartening that she can vote.’ Is that what you meant?”
Distract: ”I have a question about this patient’s condition” or “I have the patient in room “x,” is it alright if we go to their room now?
Delegate: reporting or discussing to another individual or to a reporting system
Display Discomfort: frown, avoid laughter, avoid nodding.
Delay: discuss the comment with the attending in a more private setting

## Slide 14
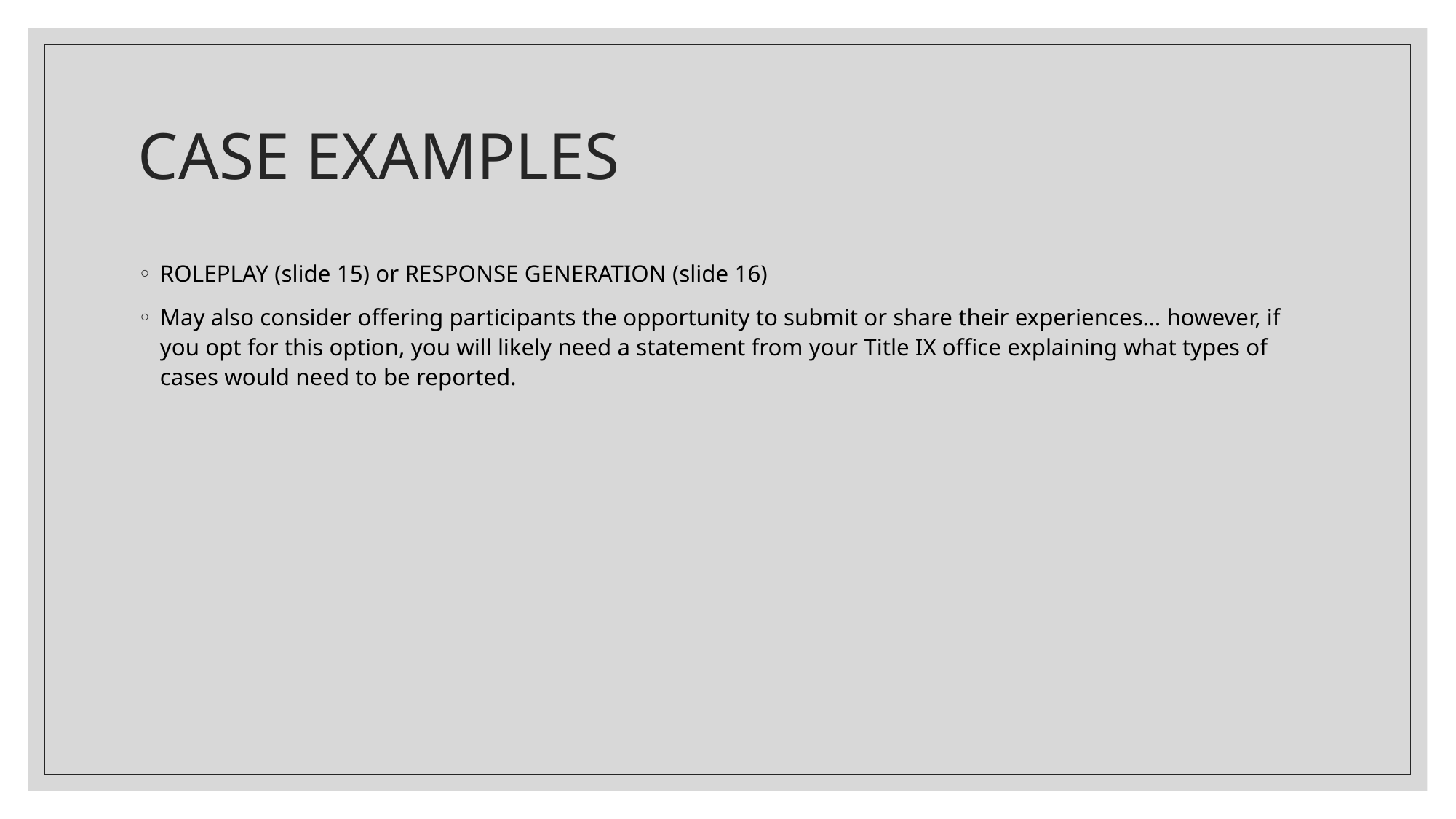

# CASE EXAMPLES
ROLEPLAY (slide 15) or RESPONSE GENERATION (slide 16)
May also consider offering participants the opportunity to submit or share their experiences… however, if you opt for this option, you will likely need a statement from your Title IX office explaining what types of cases would need to be reported.

## Slide 15
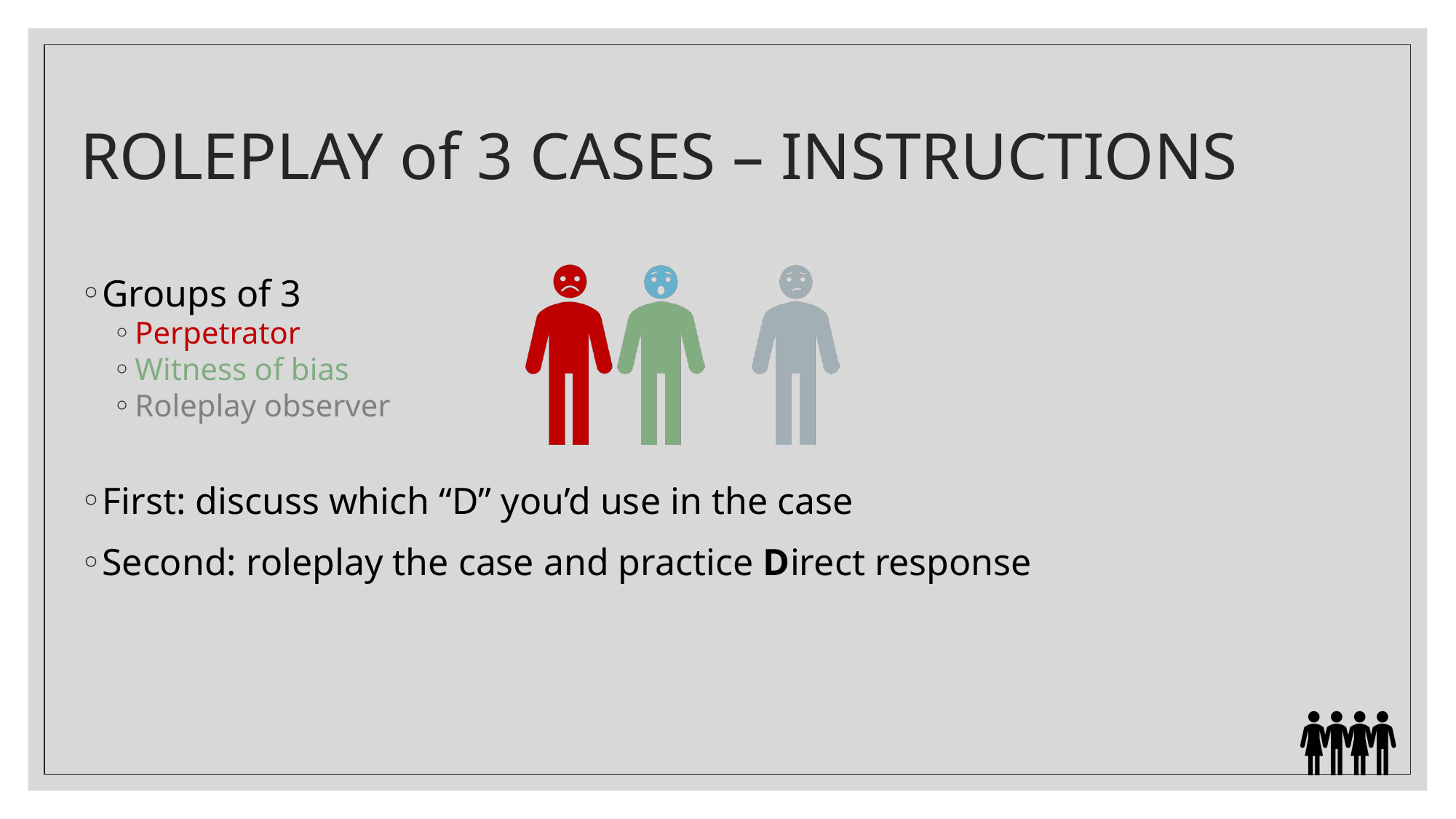

# ROLEPLAY of 3 CASES – INSTRUCTIONS
Groups of 3
Perpetrator
Witness of bias
Roleplay observer
First: discuss which “D” you’d use in the case
Second: roleplay the case and practice Direct response

## Slide 16
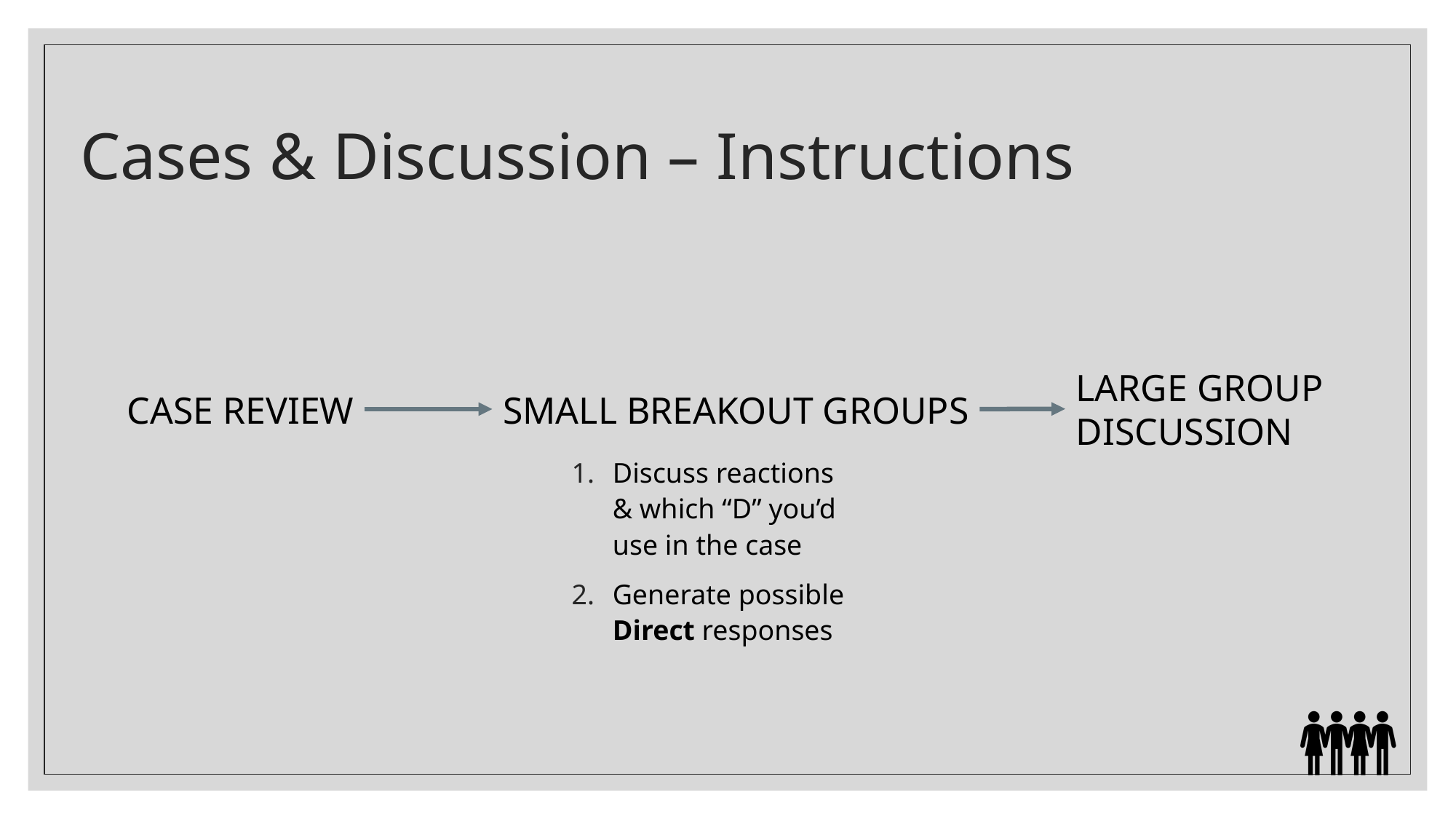

# Cases & Discussion – Instructions
LARGE GROUP
DISCUSSION
CASE REVIEW
SMALL BREAKOUT GROUPS
Discuss reactions & which “D” you’d use in the case
Generate possible Direct responses

## Slide 17
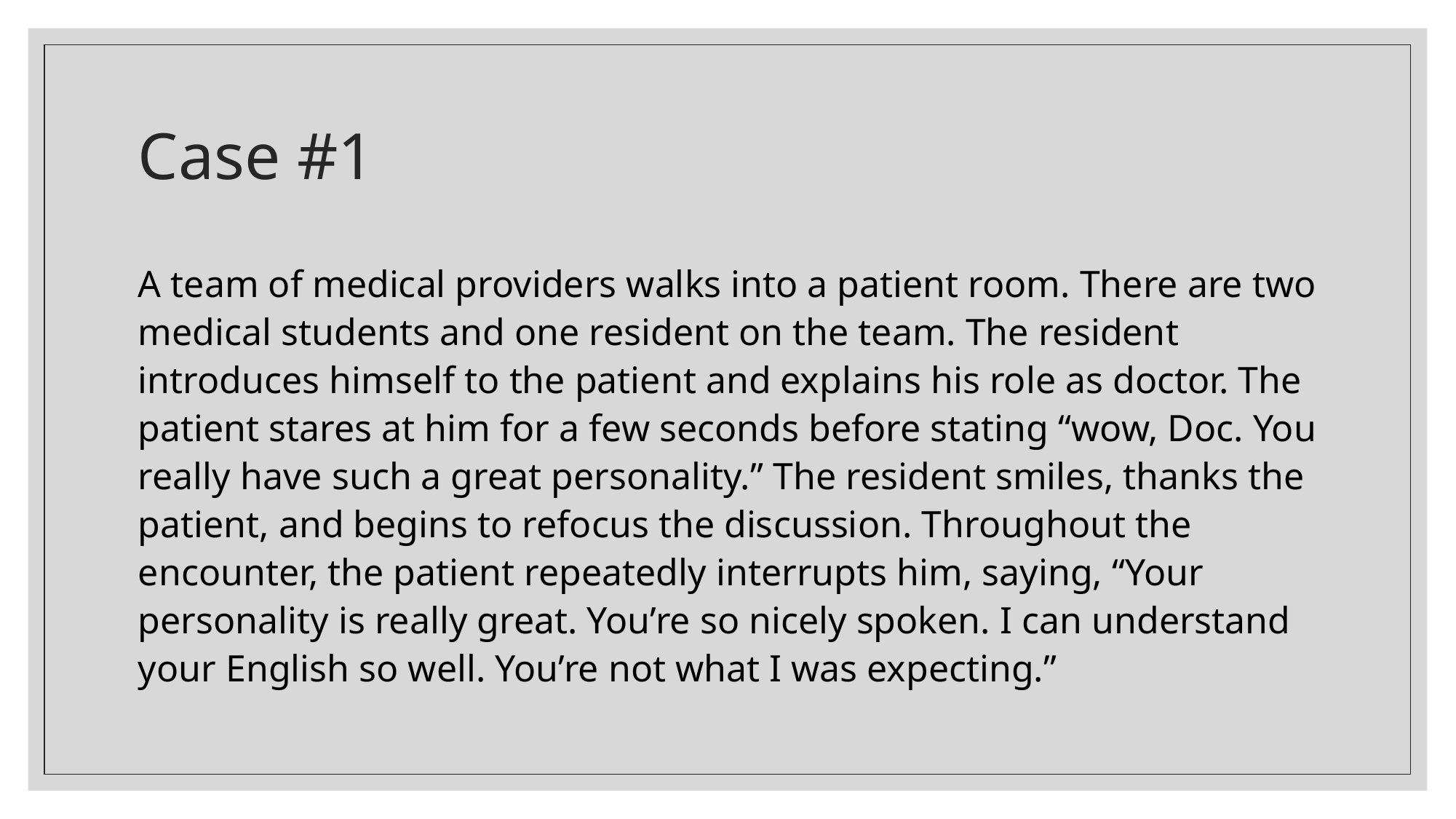

# Case #1
A team of medical providers walks into a patient room. There are two medical students and one resident on the team. The resident introduces himself to the patient and explains his role as doctor. The patient stares at him for a few seconds before stating “wow, Doc. You really have such a great personality.” The resident smiles, thanks the patient, and begins to refocus the discussion. Throughout the encounter, the patient repeatedly interrupts him, saying, “Your personality is really great. You’re so nicely spoken. I can understand your English so well. You’re not what I was expecting.”

## Slide 18
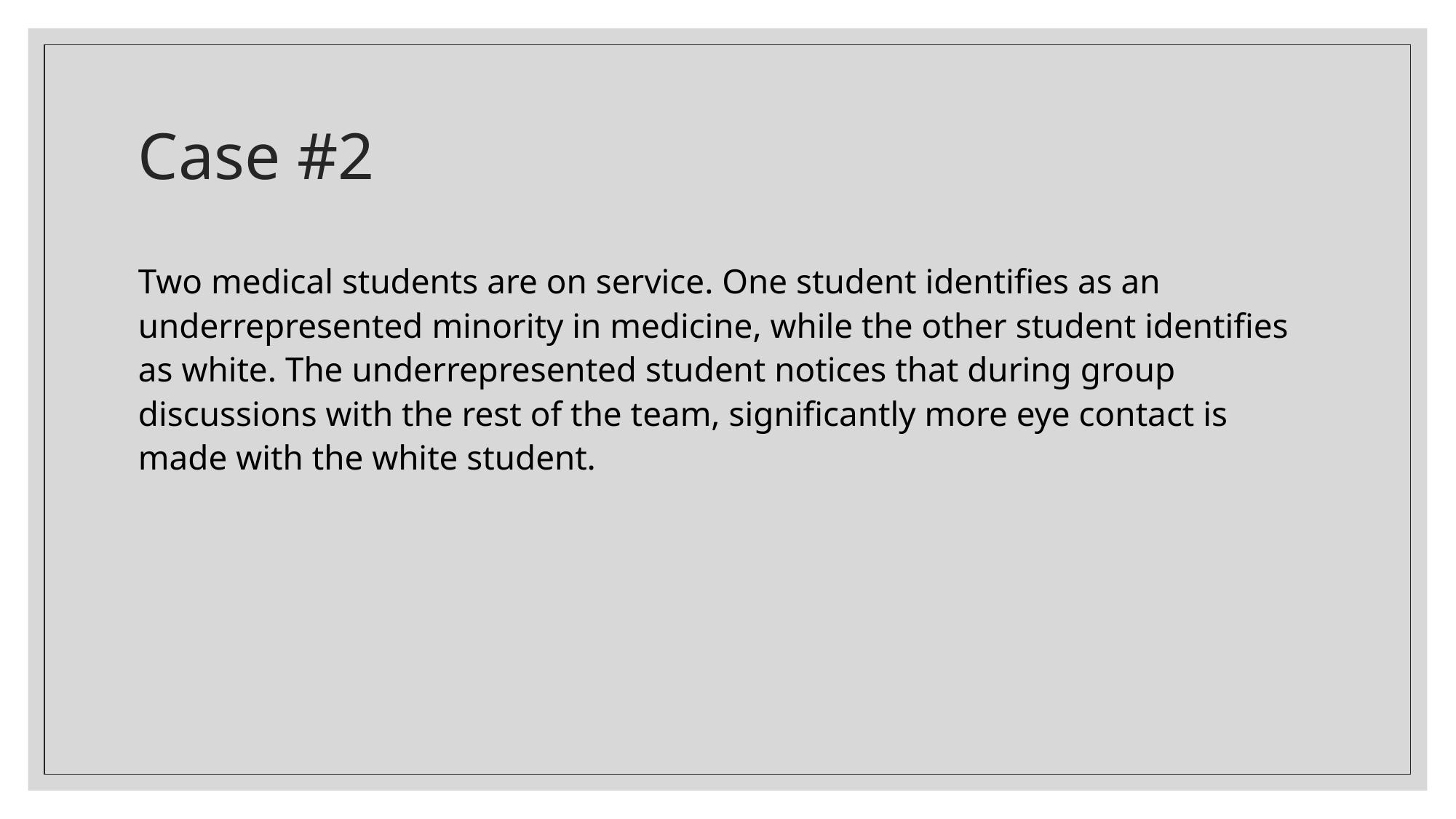

# Case #2
Two medical students are on service. One student identifies as an underrepresented minority in medicine, while the other student identifies as white. The underrepresented student notices that during group discussions with the rest of the team, significantly more eye contact is made with the white student.

## Slide 19
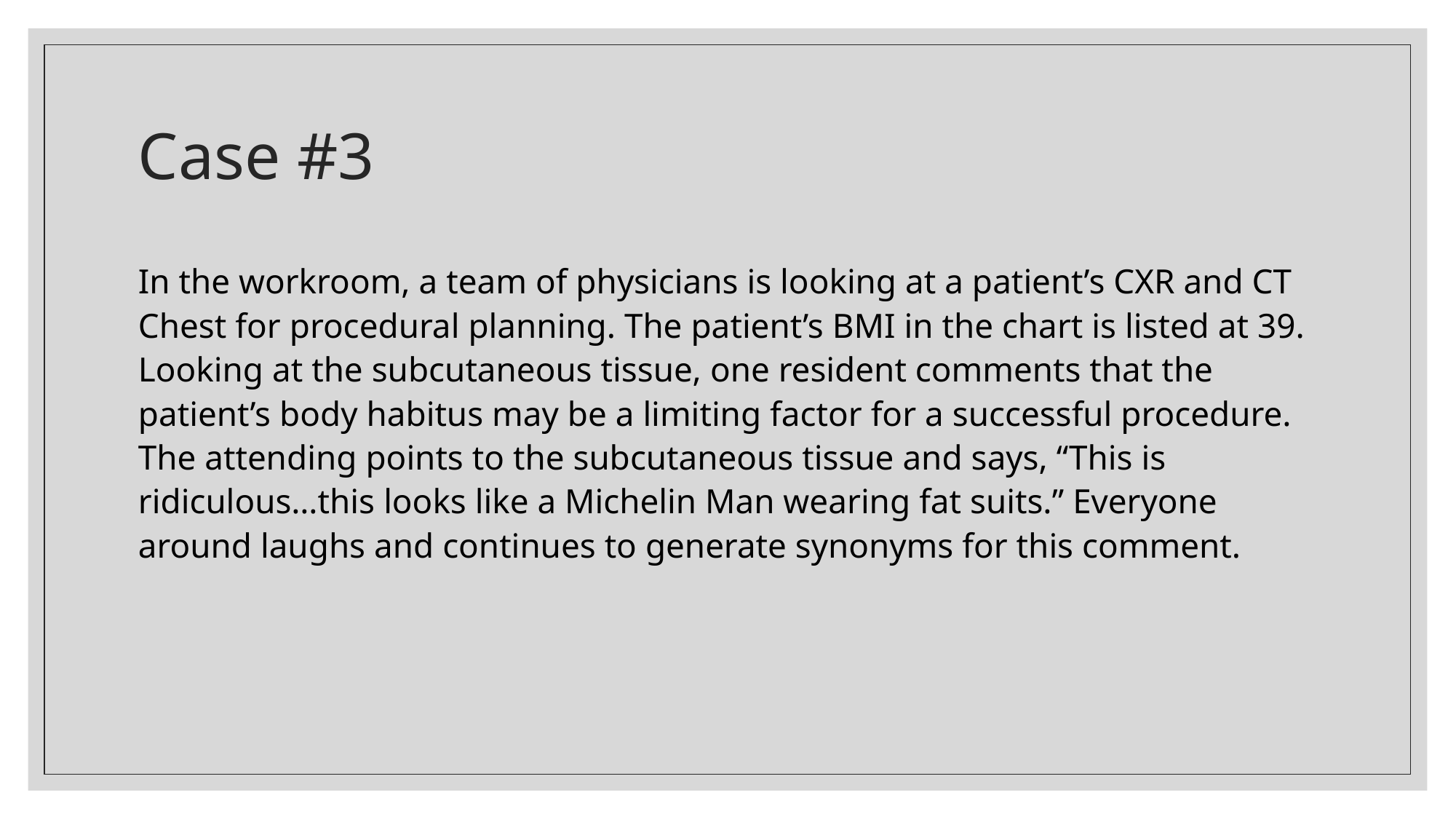

# Case #3
In the workroom, a team of physicians is looking at a patient’s CXR and CT Chest for procedural planning. The patient’s BMI in the chart is listed at 39. Looking at the subcutaneous tissue, one resident comments that the patient’s body habitus may be a limiting factor for a successful procedure. The attending points to the subcutaneous tissue and says, “This is ridiculous…this looks like a Michelin Man wearing fat suits.” Everyone around laughs and continues to generate synonyms for this comment.

## Slide 20
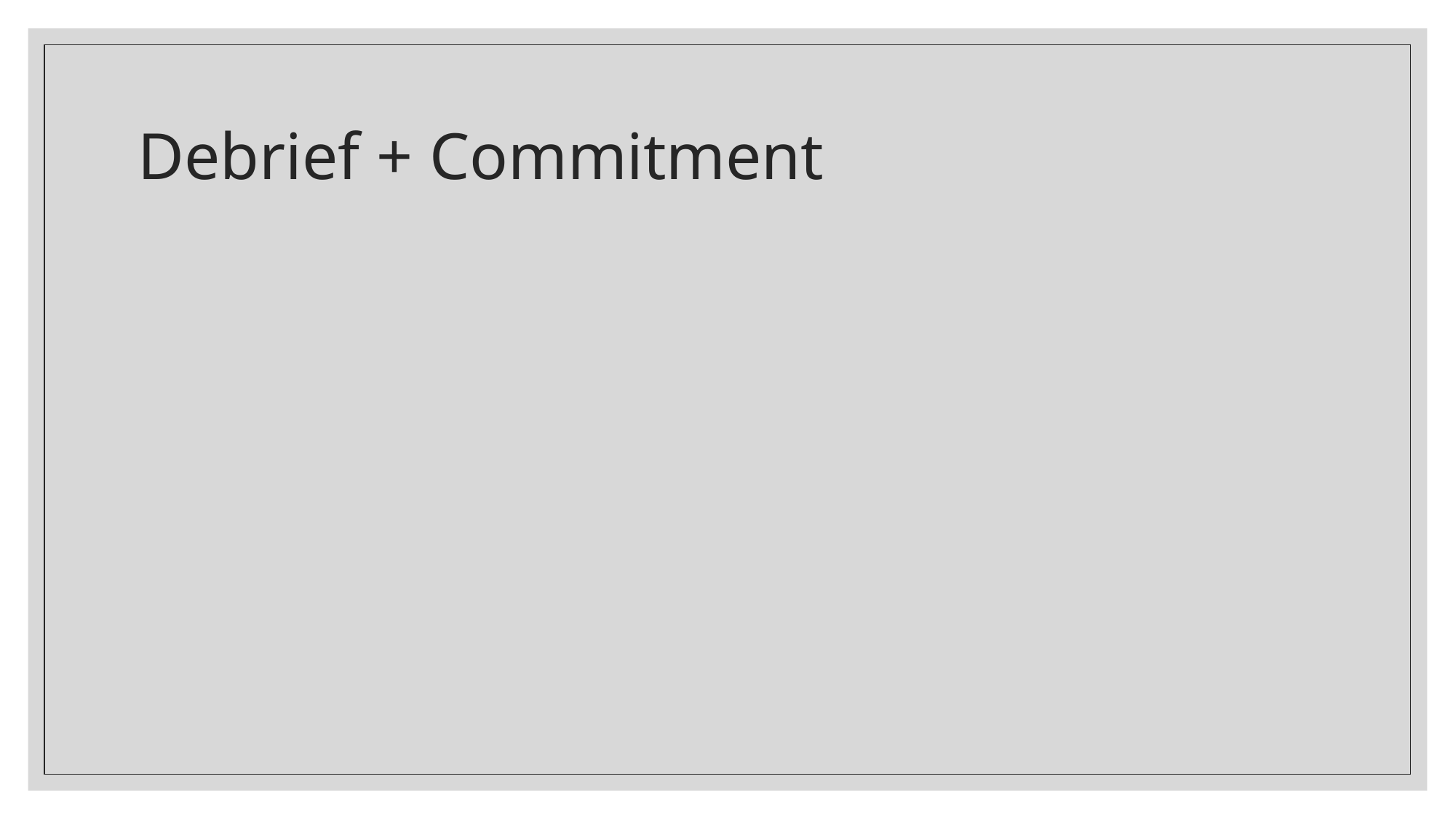

# Debrief + Commitment

## Slide 21
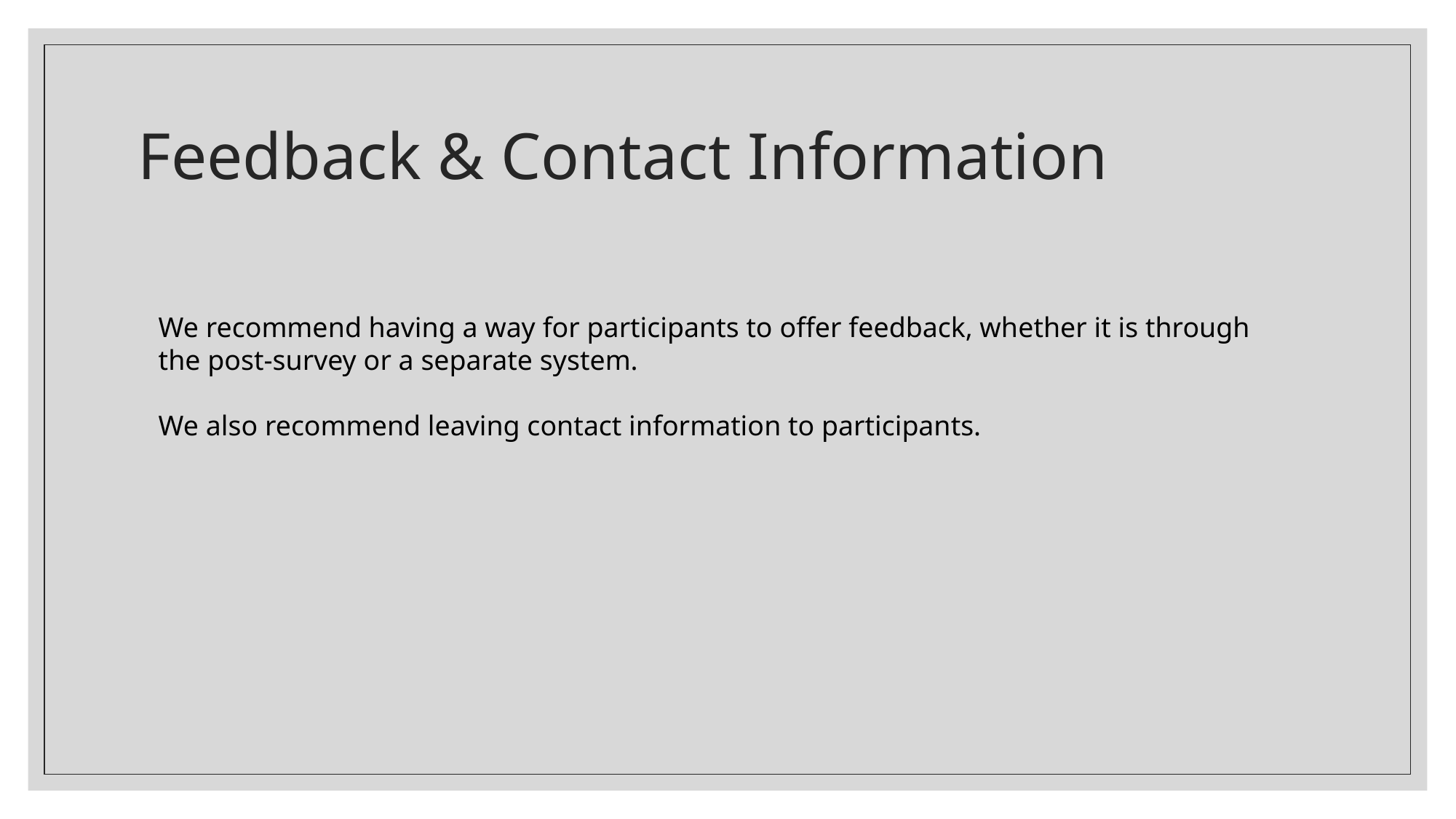

# Feedback & Contact Information
We recommend having a way for participants to offer feedback, whether it is through the post-survey or a separate system.
We also recommend leaving contact information to participants.

## Slide 22
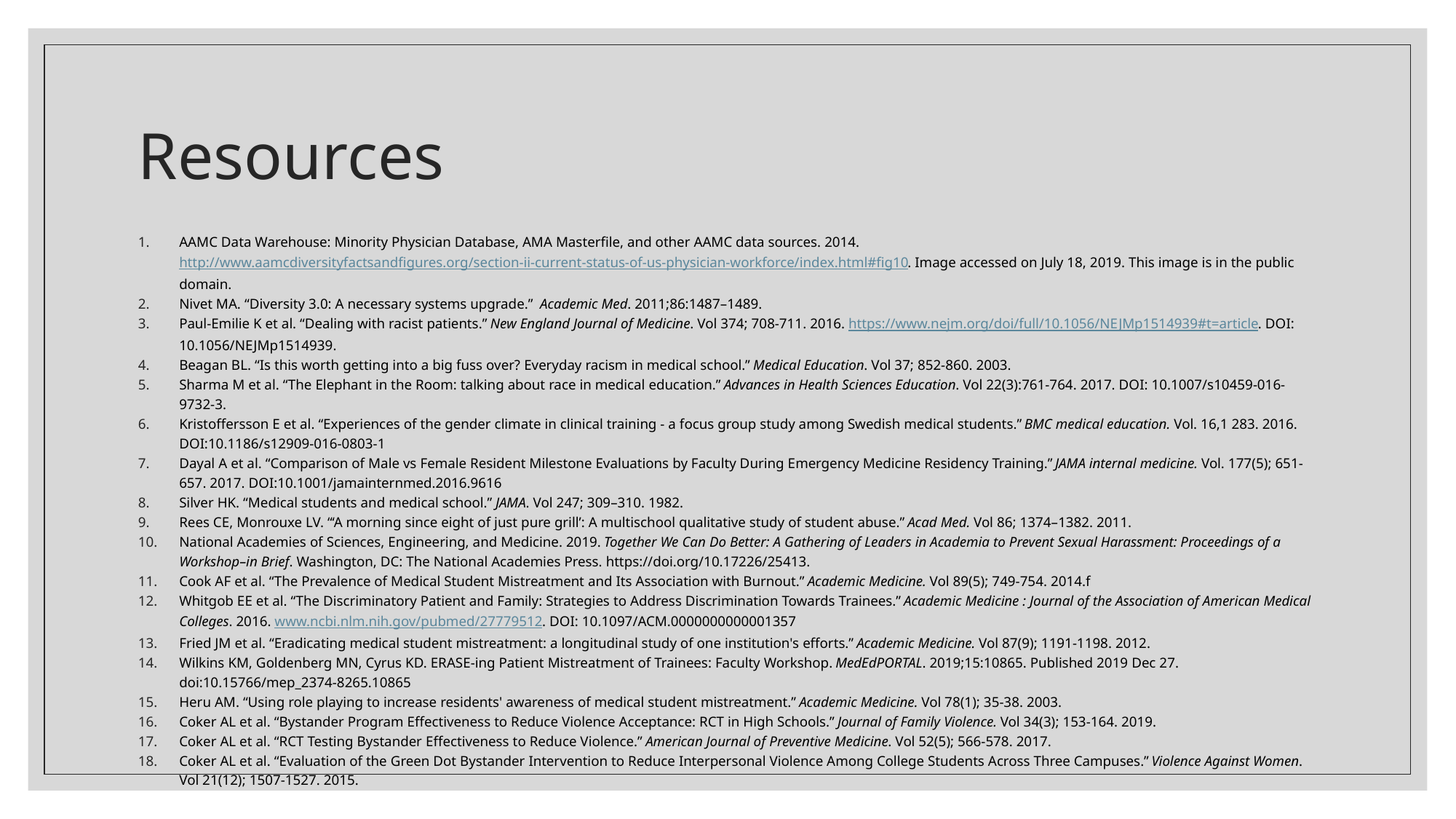

# Resources
AAMC Data Warehouse: Minority Physician Database, AMA Masterfile, and other AAMC data sources. 2014. http://www.aamcdiversityfactsandfigures.org/section-ii-current-status-of-us-physician-workforce/index.html#fig10. Image accessed on July 18, 2019. This image is in the public domain.
Nivet MA. “Diversity 3.0: A necessary systems upgrade.” Academic Med. 2011;86:1487–1489.
Paul-Emilie K et al. “Dealing with racist patients.” New England Journal of Medicine. Vol 374; 708-711. 2016. https://www.nejm.org/doi/full/10.1056/NEJMp1514939#t=article. DOI: 10.1056/NEJMp1514939.
Beagan BL. “Is this worth getting into a big fuss over? Everyday racism in medical school.” Medical Education. Vol 37; 852-860. 2003.
Sharma M et al. “The Elephant in the Room: talking about race in medical education.” Advances in Health Sciences Education. Vol 22(3):761-764. 2017. DOI: 10.1007/s10459-016-9732-3.
Kristoffersson E et al. “Experiences of the gender climate in clinical training - a focus group study among Swedish medical students.” BMC medical education. Vol. 16,1 283. 2016. DOI:10.1186/s12909-016-0803-1
Dayal A et al. “Comparison of Male vs Female Resident Milestone Evaluations by Faculty During Emergency Medicine Residency Training.” JAMA internal medicine. Vol. 177(5); 651-657. 2017. DOI:10.1001/jamainternmed.2016.9616
Silver HK. “Medical students and medical school.” JAMA. Vol 247; 309–310. 1982.
Rees CE, Monrouxe LV. “‘A morning since eight of just pure grill’: A multischool qualitative study of student abuse.” Acad Med. Vol 86; 1374–1382. 2011.
National Academies of Sciences, Engineering, and Medicine. 2019. Together We Can Do Better: A Gathering of Leaders in Academia to Prevent Sexual Harassment: Proceedings of a Workshop–in Brief. Washington, DC: The National Academies Press. https://doi.org/10.17226/25413.
Cook AF et al. “The Prevalence of Medical Student Mistreatment and Its Association with Burnout.” Academic Medicine. Vol 89(5); 749-754. 2014.f
Whitgob EE et al. “The Discriminatory Patient and Family: Strategies to Address Discrimination Towards Trainees.” Academic Medicine : Journal of the Association of American Medical Colleges. 2016. www.ncbi.nlm.nih.gov/pubmed/27779512. DOI: 10.1097/ACM.0000000000001357
Fried JM et al. “Eradicating medical student mistreatment: a longitudinal study of one institution's efforts.” Academic Medicine. Vol 87(9); 1191-1198. 2012.
Wilkins KM, Goldenberg MN, Cyrus KD. ERASE-ing Patient Mistreatment of Trainees: Faculty Workshop. MedEdPORTAL. 2019;15:10865. Published 2019 Dec 27. doi:10.15766/mep_2374-8265.10865
Heru AM. “Using role playing to increase residents' awareness of medical student mistreatment.” Academic Medicine. Vol 78(1); 35-38. 2003.
Coker AL et al. “Bystander Program Effectiveness to Reduce Violence Acceptance: RCT in High Schools.” Journal of Family Violence. Vol 34(3); 153-164. 2019.
Coker AL et al. “RCT Testing Bystander Effectiveness to Reduce Violence.” American Journal of Preventive Medicine. Vol 52(5); 566-578. 2017.
Coker AL et al. “Evaluation of the Green Dot Bystander Intervention to Reduce Interpersonal Violence Among College Students Across Three Campuses.” Violence Against Women. Vol 21(12); 1507-1527. 2015.
