# Supplementary material for: Becoming Active Bystanders and Advocates: Teaching Medical Students to Respond to Bias in the Clinical Setting
Source: MedEdPORTAL. 2021 Aug 19;17:11175. doi: 10.15766/mep_2374-8265.11175 (PMC8374028; doi:10.15766/mep_2374-8265.11175)
Supplement: Supplementary file 1 — Bystander Training.pptxFacilitator Guide.docxResponse Framework Handout.docxExample Cases.docxSurveys.docxFocus Group Facilitator Guide.docx [file mep_2374-8265.11175-s001.zip › B. Facilitator Guide.docx]

**Medical Student Bystander Training Workshop: Facilitator Guide**

Authors: Michelle York BS, Amy E Fleming MD MHPE

Vanderbilt University School of Medicine

# Total session time: 55 min

# Ideal number of participants: 12-18

# (See below for suggested modifications if given a shorter time frame and larger audience.)

**Educational Objectives:**

1. Define the terms bias, microaggression, and active bystander.
2. Summarize the prevalence of bias and microaggressions.
3. Describe the 5 D's model as a framework to respond to incidents of bias and microaggressions as an active bystander
4. Explain the importance of active bystander advocacy as a model for improving the clinical learning and care environment
5. Generate possible responses to example cases of bias or microaggressions

# Materials

# PowerPoint slide presentation (adapted)

# Discussion cases (or see point 4. below)

# “5 D’s Framework” handout

# Pre- and Post-Workshop Surveys

| **PRE-SESSION** |
| --- |

Adapt slides to your institution

1. Consider surveying group during workshop to gauge perceptions using tools such as Poll-Everywhere
2. Be sure to identify resources for “delegate” – i.e. what are the appropriate resources that participants can report to or use if they experience an incident?
3. Consider who will be giving the training. At the Vanderbilt University School of Medicine, our students responded positively to having the event led by both a student and administrator. The administrator, a Dean in our case, helped show that the administration was invested in the workshop, while the student helped to create a peer-to-peer discussion setting.
4. Consider your case examples. You may want to talk to participants and see if your example cases capture some of their experiences. This is not meant to be an all-encompassing list, but should include a variety of scenarios. We recommend including cases of microaggressions, as this helps participants to practice 1) identifying microaggressions, and 2) responding to these more “subtle” incidents. Further, you may want to consider contacting your **Title IX office** if you’d like to have participants submit experiences prior to or share experiences during the workshop. This was a suggested area for improvement for our team. The Title IX office may be able to help give you an encompassing statement that discloses what types of cases you would need to report, which would help transparency and safety.

| **INTRODUCTION + PRE-SURVEY** *– 3m* |
| --- |

- If you are conducting a pre-survey, begin by administering your questionnaire or our Pre-workshop survey
- Introduction
- Objectives & timeline
- Overview of expectations

**SLIDE 1: INTRODUCTION**:

- Hello and welcome to the Bystander Training workshop! Briefly share with your participants why you’re there to speak with them and offer a broad overview of what you hope the group will accomplish during the session
  - Example: *You all are here because our school of medicine and medical center recognize that bias and microaggressions are real issues that are prevalent in medical communities, including our own. We also know that most physicians and students struggle with how to respond to forms of mistreatment. Our administration wants you to be better prepared to help promote positive change in our culture by giving you some tools to help you respond to these incidents.*

**OVERVIEW OF EXPECTATIONS**

- Because this discussion may bring up difficult subjects, be sure to expectations on how participants should participate and interact with one another. Set guidelines necessary (e.g. do not ask others to speak on behalf of a group, etc.) Instruct participants to be respectful of one another in order to create space for participation.
  - Example: *We know that not everyone in this room experiences bias or microaggressions in the same way. Some of you, particularly those of you who identify with underrepresented social demographics, may experience this much more frequently than others. That said, whether you are targeted or you are the witness, I want to be clear that this is everyone’s problem, and it is everyone in this room’s responsibility to stand up and speak up to stop this from perpetuating. Furthermore, it is everyone’s responsibility to do the same for your patients and your team members whose professions hold “less” authority. So, please be respectful of your peers today and approach today’s session with a lens of learning. Please do not ask others to speak on behalf of a group, but create an environment of respect so that we can allow different perspectives to be heard.*

**SLIDE 2 & 3: OBJECTIVES & TIMELINE**

- Review the Objectives of the session (see above)
- Timeline: Set expectations of your timeline for participants so that you can effectively manage the session. Depending on the length of your workshop, adjust your timeline as needed.
  - Example: *our workshop was 55-minutes*
    - *Pre-survey – 3 minutes*
    - *Introduction – 3 minutes*
    - *Background information + group Poll Everywhere – 6 minutes*
    - *Framework: 5 D’s – 8 minutes*
    - *Case examples + practice – 28 minutes*
    - *Debrief + post-survey – 8 minutes*

| **BACKGROUND INFORMATION** *– 6m* |
| --- |

- Introduction to terminology
- Background data

**SLIDE 4: INTRO TO TERMINOLOGY**

- Before jumping into the framework and cases, it is important to ensure that participants have a shared definition for and understanding of the terms being used
  - *In our workshop, we asked students to share their definitions with us before revealing our definition. This allowed us to get a sense of where the group’s understanding was so that we could adjust how much time was spent discussing each term. One tip – in a quieter group you may ask participants to start by “pair and share” their definitions with the person sitting next to them. Then, return to the large group and ask someone to share what they discussed. This can help people feel more confident and comfortable sharing.*
- **Bias**: a prejudice in favor of or against one thing, person, or group compared with another, usually in a way that is **clearly unfair** to the observer or victim.
  - **Explicit** **bias**: conscious awareness of attitudes or beliefs
  - **Implicit** **bias**: unconscious prejudices; beyond the individual’s awareness or intentional control
  - It is important to note that while we are making the distinction between implicit and explicit bias for your understanding, when witnessing or experiencing an incidence of bias or a microaggression, we encourage you to respond based on the consequence of the action or interaction, rather than responding based on the intent.
- **Microaggression**: a statement, action, or interaction regarded as ***indirect or subtle****,* often unintentional, discrimination or prejudice against a specific demographic.
  - *A great analogy to share with participants is the theory posed by Dr. Valencia Walker from UCLA. Dr. Walker suggests that Microaggressions are like Mosquito bites. This theory can be helpful as it nicely addresses the fact that they are often subtle, but can be accompanied by significant discomfort, annoyance, and even pain for the affected individual.*
  - *Optional: You may consider using the following video as an aid for demonstrating this principle:* [*https://www.youtube.com/watch?v=emz49wSnNcs*](https://www.youtube.com/watch?v=emz49wSnNcs)
  - *Another important point to note is that, while the concept of microaggressions is well established, the prefix “micro” can undermine the significant impact and distress that these interactions can cause for the targeted individual or victim. Thus, while “micro” refers to their subtlety, it should not be confused with the emotional or psychosocial impact on the individual.*
- **Active bystander** (also termed “Upstander”): someone who not only witnesses a situation, but takes steps to intervene or address the situation in a way that prevents further escalation of the issue or harm to the victim
  - *For medical groups, you can easily relate this concept to an example they are already familiar with: how to respond when a patient is in cardiac arrest. This is a common example even for non-medical personnel. The active bystander in that situation is a person who intervenes by calling 9-1-1, initiating CPR, calling a code, etc.*
  - *The concept of being an active bystander can therefore be applied in cases of biases or microaggressions.*
  - *Something you may want to acknowledge is that* ***not everyone wants to be “rescued”*** *from a situation. Participants may offer this as something they’ve struggled with knowing how to identify after witnessing a situation.*

**SLIDES 5-7 (OPTIONAL): POLL YOUR CROWD**

- *After reviewing those definitions with your participants, you may want to consider gathering their thoughts on the prevalence and importance of addressing these issues. As everyone in the room has different experiences with these incidents, and some experience it much more frequently, this can help the group to realize many people have experienced or witnessed these incidents. This helps participants feel less alone, and may make them more engaged in the discussion or sharing.*

**SLIDE 8: BACKGROUND DATA**

- Review the important background data on the prevalence/impact of microaggressions and bias, particularly on under-represented groups in medicine.
  - Data published by the AAMC shows that over the past thirty years, the demographic makeup of graduating physicians has shifted dramatically, with the number of racial minorities and women increasing significantly.^1^


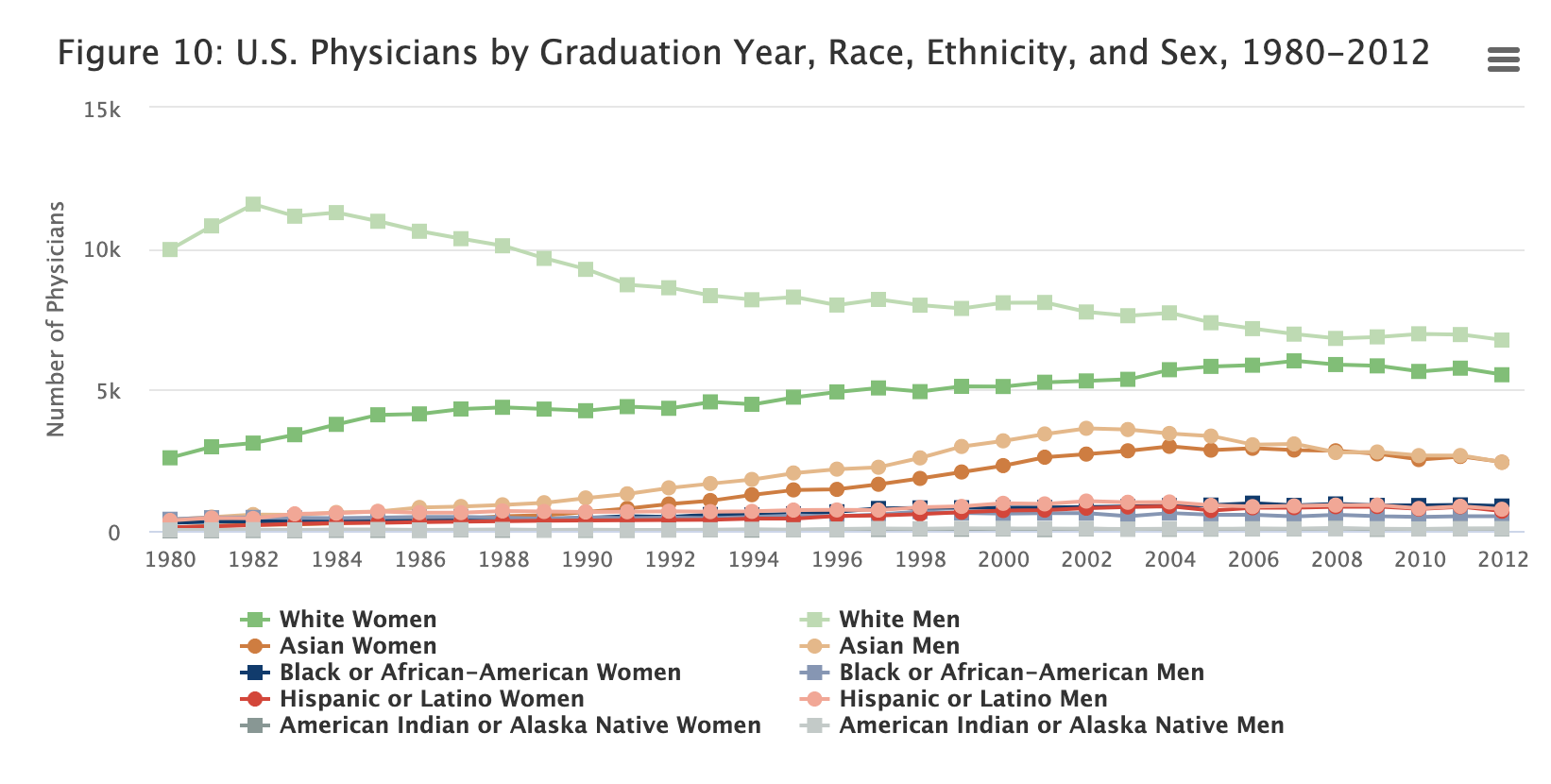


The above image by the AAMC was retrieved from [http://www.aamcdiversityfactsandfigures.org/section-ii-current-status-of-us-physician-workforce/index.html#fig10](http://www.aamcdiversityfactsandfigures.org/section-ii-current-status-of-us-physician-workforce/index.html) on July 18, 2019. This Image is in the public domain.

- - - *You may want to acknowledge that the medical community still has a long journey before the physician profession demographically reflects or is representative of the population they serve.*
  - Improved diversity of medical providers has improved patient care.^1^ Despite this, under-represented minorities, women, and medical students continue to experience substantial bias, which can lead to burnout, limit the culture of inclusivity, and impact career advancement opportuntiies^2-11^
    - National Academy of Sciences report: female medical students and gender harassment^10^
      - *This study identified that female medical students experienced more gender-based harassment at higher rates than students in other fields. Note that gender-based harassment were instances of belittling individuals based on their gender identity.*
    - Prior published strategies have had limited success^12^ or have been targeted towards improving faculty or resident responses.
      - *While the prior approaches have been excellent in their methodology, many have shown limited impacts on reducing mistreatment. Further, many were focused on training individuals in residency or faculty positions. While these are excellent, we think that our training offers an opportunity to adaptable training framework that can be adopted by students, residents, faculty, nurses, staff, etc.*
  - Acknowledge that the clinical hierarchy sets up medical students to be subjected to mistreatment, as within the physician training hierarchy, they are at the “lowest rung.”
    - *You may want to take a moment to re-frame this, as although medical students are considered to be the lowest in the MD training hierarchy, the pursuit of an MD already grants them favorable positions and presumptions based on that undertaking. They are often presumed to be smart, motivated, etc. Thus, medical students may have more “power” than other groups and professions in the hospital, giving students a responsibility to speak up.*

**SLIDE 9: RESPONSIBILITY**

- We used the next slide as an opportunity to broaden the scope of the discussion and set the stage for why learning these skills *early* is important.
  - *Example: Translating what we’ve just discussed into the broader setting of society, your decision to go into medicine, right or wrong, grants you a favorable position in the broader community, which gives your voice elevated weight or authority. With that, we empower you to accept the responsibility that comes with this status to serve as advocates for your classmates, medical teams, patients, and community members.*
- Today we’re going to talk about different strategies you can take to using your voice to effect change.

| **FRAMEWORK: 5 D’s** *– 8m* |
| --- |

**SLIDE 10: GREEN DOT BACKGROUND**

- Introduce the foundation for the framework: Green Dot & Dr. Kimberly Manning’s modifications:
  - Green Dot is a widely used educational program that focuses on reducing power-based mistreatment through training active bystanders. It was originally developed at the University of Kentucky to target sexual violence on college campuses and Randomized Control Trials have demonstrated its efficacy in reducing interpersonal violence.
    - It has also been applied to additional settings, such as workplaces and high schools.
    - It’s training model is based around the mnemonic the 3 D’s, which we will describe next.
  - Using this as a model, Dr. Kimberly Manning, Professor of Medicine at Emory University, has since expanded this structure to include two additional D’s.
  - This framework can be applied to incidents of experienced OR witnessed microaggressions or bias.
  - We’re going to walk through each of these response types together.

**SLIDE 11-13: FRAMEWORK DISCUSSION**

- Introduce the 5 D’s framework, referring to the handout and slides to do so. Provide participants with definitions as well as examples.
  - *You may want to use the example listed on the handout, or the one listed on slide 13. This applies the framework to a case and gives participants the chance to see what “D” they may feel comfortable using. This also demonstrates to participants that this is a dynamic and adaptable framework.*

| **CASE EXAMPLES + PRACTICE** *– 30m* |
| --- |

**SLIDE 15 or 16: CASE INSTRUCTIONS**

- *Slide 14 details a few options to consider for how you want your participants to approach the cases. We have detailed two methods that we have tried – roleplay and response generation – and both have pros and cons. Roleplay can be a great way to try to practice direct responses, but also has the caveat of not being realistic if participants are amongst their peers. Response generation may identify possible responses, but people may not feel as practiced coming out of the session. We have outlined suggestions for both options below.*
- You may want to select your cases ahead of time. We have included a list of example cases in the Appendix.
  - Please see “Session Modifications” section below for additional considerations.
- When introducing the cases, acknowledge that the cases may oversimplify the interactions and that your selected scenarios are not meant to be an exhaustive list of problematic comments or situations.
- ROLEPLAY:
  - Divide your participants into groups of 3
  - After reading the case together, have them discuss in their breakout groups what “D” they think they would most likely use.
  - Second, have participants roleplay in their breakout groups to practice the “Direct” responses, as these tend to be the most difficult. For each case there will be a “perpetrator,” a responder, and an observer. Ask members to rotate which role they play for each case.
  - *To help participants easily adopt the roles and focus on the task, we recommend clarifying from the case exactly who the perpetrator is and who the responder is.*
- RESPONSE GENERATION:
  - Divide your participants into groups of 3-5.
  - After reading the case together, have them discuss in their breakout groups what “D” they think they would most likely use.
  - Second, have them think of different responses that they could use within the Direct, Delay, or Distract category.
    - *You may ask them to keep notes or to submit these ideas to server such as Poll Everywhere. We do recommend that someone present takes notes during the large group discussion so that you can give participants a list of the responses generated from the session. Our participants found this to be very helpful and provided a tangible, useful skill.*
- Return to the large group for discussion and reflection on what was identified or experienced in the small groups.
  - *If your total participant number is small enough, you may be able to go group-by-group in this to ensure you hear from everyone.*
- TIMING:
  - For either scenario, we recommend spending ~10 minutes per case.
    - To achieve this, we had students spend ~4 minutes in small groups and ~6 in large group discussion.

| **DEBRIEF + POST-SURVEY**– *8m* |
| --- |

- Facilitate a debrief from the discussion.
  - The conversation may be challenging. Allow space for participants to share their reflections on the case or on their own experiences.
  - Prompting questions:
    - Did groups tend to gravitate towards using one “D” in particular? If so, why did it feel most comfortable and why did the other response options not afford the same comfort level?
      - Did it vary based on the position of the microaggressor?
    - Were you surprised by the cases? How did these cases compare to what you have seen or experienced?
    - Have there been times where you or someone else has successfully intervened?
    - We recognize that this can feel overwhelming to take on… so what is ONE piece that you feel that you can take on? What is one thing you are committed to trying?
    - What are some possible limitations to this framework? Where are the gaps in its application?
- Summarize the 5 D’s framework for them again
- Consider providing a list of resources where they can delegate or debrief after an experienced or witnessed situation.
- Consider administering the post-survey.

| **SESSION MODIFICATIONS** |
| --- |

- **Group size:** While we recommend working in groups of 12-18, we first conducted this session in a group of 100 students. It can certainly be applied to large settings, but the selection for how to work through the cases and how to manage the large group debrief after each case will need to be adjusted. We advised 12-18 participants as, based on feedback from a focus group, this was the setup that felt most comfortable for sharing and productive discussion.
- **Virtual formats:** We adapted this session to Zoom during the COVID-19 Pandemic. The main adjustments were as follows:
  - For polling, we used Zoom’s polling feature instead of adding in additional software
  - We copied the cases into the text box to limit having to switch back and forth into screen-sharing.
  - We used breakout groups for small-group discussions. Be sure to give them a warning when they have about ~1-minute remaining in their small group.
- **Cases:** We have provided example cases in the appendix. As discussed above, you may decide that you’d prefer your participants submit their experiences prior to or share their experiences *during* the workshop. We received a lot of feedback requesting this method, as many participants wanted to bring forward cases they had seen that they struggled with. If you wish to pursue this route, you will likely need to contact your institution’s **Title IX office**. Their team may be able to help give you an encompassing statement that discloses what types of cases you would need to report, which would help transparency and safety.
