# Supplementary material for: Becoming Active Bystanders and Advocates: Teaching Medical Students to Respond to Bias in the Clinical Setting
Source: MedEdPORTAL. 2021 Aug 19;17:11175. doi: 10.15766/mep_2374-8265.11175 (PMC8374028; doi:10.15766/mep_2374-8265.11175)
Supplement: Supplementary file 1 — Bystander Training.pptxFacilitator Guide.docxResponse Framework Handout.docxExample Cases.docxSurveys.docxFocus Group Facilitator Guide.docx [file mep_2374-8265.11175-s001.zip › C. Response Framework Handout.docx]

**5 D’s Response Behavior Framework**

| The 5 D’s model was developed by Dr. Kimberly Manning from Emory University School of Medicine. With her permission, our curriculum was expanded from the 3 D’s of the Green Dot program (Direct, Delegate, and Distract) to the 5 D’s, which are described below.  Keep in mind: there is no universal way to respond or superior response type. These are all valid ways to take meaningful action and contribute to promoting a culture of safety. You may adjust your response selection based on the incident severity, your relationship with the individual, the setting and your company, and many other factors. This is not about being perfect, but about trying to contribute to a safe patient care and clinical learning environment. |
| --- |

**Direct:** verbally respond to the perpetrator in the moment.

- We are trained to observe and describe or report those observations. You may consider incorporating this skill into the stepwise approach below:
  - Step 1: Objective – state the facts of what you heard or saw.
  - Step 2: Subjective – share your interpretation and how this affected you or made you feel.
  - Step 3: Listen – be open to hearing what the other person has to say

**Distract:** defuse the situation by shifting the attention or focus of the perpetrator to prevent further harm.

- Medical students/learners are often well-positioned for this approach, as you may try shifting the conversation back to a learning point.
  - Can we discuss how you would care for this patient? Can we review the mechanism of that drug?

**Delegate:** take action by delegating this responsibility to someone who may be able to better approach the individual and engage them in discussing the incident.

- This may be used in situations where you do not feel safe or comfortable responding to the individual. In this case, by relaying what you witnessed or heard, the individual can approach the perpetrator for a discussion.
- Examples of who you can delegate to:
  - Individuals: Course director, Attendings, Residents, School Deans, Program directors, mentors.
  - Institutional reporting systems

**Delay:** discuss the situation with the perpetrator or victim later.

- This can give you 1) time to process the event, and/or 2) a more private setting for your conversation to take place.
- You can apply the same steps as described in the “direct” response.

**Display Discomfort:** exhibit discomfort or concern in response to the incident.

- This can be used in situations where you do not feel comfortable verbally responding, or when you want to supplement your verbal response. It can be an effective way to communicate your disagreement with the situation.
- To do this, you may frown, furrow or raise your eyebrows, or keep a straight face.

If possible, avoid behaviors such as nodding or laughing, as these can provide positive affirmation the individual.

**EXAMPLE CASE:**

A patient says to a provider, “I’ve had great care at this hospital, except for when “foreign” people care for me. They’re loud and rude.”

1. DIRECT:

*“I’m sorry you have had a negative experience with an individual in the past. Great medical care is a team effort. You’ve got a wonderful group of individuals from a diverse array of backgrounds working hard to make sure you receive the care you need. I assure you they are all exceptionally qualified.”*

*“Our expectation is that both our staff and patients are treated with respect. We ask that you uphold that expectation, and we will work to ensure that we do the same.”*

*“I’m disappointed to hear this. Our team is comprised of many individuals from different backgrounds and this diversity of perspectives and training experiences is what makes us great.”*

2. DISTRACT:

*“I’d like to ask you more about your symptoms. Can you tell me about…”*

3. DELEGATE: Share your observations to your attending and alert them that this behavior was exhibited.

4. DISPLAY DISCOMFORT: frown or display disappointment

5. DELAY:

*“Earlier you made a comment to me about the care you’ve received here in the past. I heard you say that you’ve “had great care at this hospital, except for when ‘foreign’ people care for [you].” I wanted to address this with you. Our team is comprised of many individuals from different backgrounds, and I assure you we are all qualified and committed to providing you with exceptional care. I’m sorry you’ve had a negative experience in the past, but I have worked with these individuals and they are wonderful people deserving of your trust and respect.”*
