# Supplementary material for: Becoming Active Bystanders and Advocates: Teaching Medical Students to Respond to Bias in the Clinical Setting
Source: MedEdPORTAL. 2021 Aug 19;17:11175. doi: 10.15766/mep_2374-8265.11175 (PMC8374028; doi:10.15766/mep_2374-8265.11175)
Supplement: Supplementary file 1 — Bystander Training.pptxFacilitator Guide.docxResponse Framework Handout.docxExample Cases.docxSurveys.docxFocus Group Facilitator Guide.docx [file mep_2374-8265.11175-s001.zip › D. Example Cases.docx]

**BYSTANDER TRAINING EXAMPLE CASES**

**CASE 1:**

Two medical students are on service. One student identifies as an underrepresented minority in medicine, while the other student identifies as white. The underrepresented student notices that during group discussions with the rest of the team, significantly more eye contact is made with the white student.

**CASE 2**:

In the workroom, a team of physicians is looking at a patient’s CXR and CT Chest for procedural planning. The patient’s BMI in the chart is listed at 39. Looking at the subcutaneous tissue, one resident comments that the patient’s body habitus may be a limiting factor for a successful procedure. The attending points to the subcutaneous tissue and says, “This is ridiculous…this looks like a Michelin Man wearing fat suits.” Everyone around laughs and continues to generate synonyms for this comment.

**CASE 3:**

A Black Vanderbilt medical student is joining a new clinical service. When introducing himself as the medical student, he is asked if he is a visiting student from Meharry Medical College. This has happened on multiple services.

**CASE 4:**

A team of medical providers walks into a patient room. There are two medical students and one resident on the team. The resident introduces himself to the patient and explains his role as doctor. The patient stares at him for a few seconds before stating “wow, Doc. You really have such a great personality.” The resident smiles, thanks the patient, and begins to refocus the discussion. Throughout the encounter, the patient repeatedly interrupts him, saying, “Your personality is really great. You’re so nicely spoken. I can understand your English so well. You’re not what I was expecting.”

**CASE 5:**

As a resident is explaining a procedure to a patient, he mentions to the patient that his colleague, the medical student, will be present during the procedure. "I hope it won’t be too distracting for you because she’s so young and pretty. She’s really very bright.”

**CASE 6:**

A team of medical providers is rounding outside a patient’s room. The patient’s television is visible from the door, and the resident sees a conservative news channel is playing on the screen. Instead of listening to the presentation, she turns to her co-resident and says, “I’m actually going to go put in some orders. I’ll catch you at the next room.” Later in the team room, the same resident says to you, “I’m all about efficiency on rounds. My strategy is to go put in orders when we get to a patient I don’t want to have to deal with.”
