# Supplementary material for: Becoming Active Bystanders and Advocates: Teaching Medical Students to Respond to Bias in the Clinical Setting
Source: MedEdPORTAL. 2021 Aug 19;17:11175. doi: 10.15766/mep_2374-8265.11175 (PMC8374028; doi:10.15766/mep_2374-8265.11175)
Supplement: Supplementary file 1 — Bystander Training.pptxFacilitator Guide.docxResponse Framework Handout.docxExample Cases.docxSurveys.docxFocus Group Facilitator Guide.docx [file mep_2374-8265.11175-s001.zip › E. Surveys.docx]

**PRE-WORKSHOP, POST-WORKSHOP, and LONGITUDINAL SURVEYS**

This supplementary document contains 3 sample surveys that can be used in your institution’s program. The first two surveys were designed to be administered immediately before (pre-) and after (post-) the workshop. The longitudinal survey may be administered after the workshop has been completed and participants have been able to go about their usual clinical duties. This survey may help identify if participants whether the impacts of the training are long-lasting and effective in impacting participants’ knowledge, confidence, and/or response behaviors.

**PRE-WORKSHOP SURVEY**

For **questions 1-5**, please place a check or ‘x’ in the box that best characterizes your views on each statement.

|  | Strongly Agree | Agree | Neutral | Disagree | Strongly Disagree | I don’t know |
| --- | --- | --- | --- | --- | --- | --- |
| 1. I feel safe in the clinical learning environment at [YOUR INSTITUTION]. |  |  |  |  |  |  |
| 1. Microaggressions are an issue in the clinical learning environment at [YOUR INSTITUTION] that should be addressed. |  |  |  |  |  |  |
| 1. Microaggressions are prevalent in the clinical learning environment at [YOUR INSTITUTION]. |  |  |  |  |  |  |
| 1. Bias is an issue in the clinical learning environment at [YOUR INSTITUTION] that should be addressed. |  |  |  |  |  |  |
| 1. Bias is prevalent in the clinical learning environment at [YOUR INSTITUTION]. |  |  |  |  |  |  |

1. In the clinical learning environment, how often have **you experienced** biases and/or microaggressions? Please provide your best estimate.
   1. Never
   2. A few times
   3. Monthly
   4. Weekly
   5. Daily
   6. Other (please describe):
2. If you have **experienced** biases and/or microaggressions, did you respond to the situation?
   1. Yes
   2. No
3. If you did not respond, why? (select all that apply)
4. The hierarchy and power dynamics made me feel that I should not respond.
5. I was worried about grade or other academic consequences
6. I was worried about personal consequences
7. The instance didn’t seem important enough
8. I did not want the perpetrator to get in trouble
9. I knew the perpetrator, and I did not think this behavior was in-character
10. I was not sure if I interpreted the situation appropriately
11. Other:
12. If you did respond, how did you address the individual(s) involved? Please place a check or ‘x’ in the box that best characterizes your views on each statement.

|  | Always | Often | Sometimes | Rarely | Never |
| --- | --- | --- | --- | --- | --- |
| *I displayed discomfort or concern.* |  |  |  |  |  |
| *I addressed the perpetrator immediately.* |  |  |  |  |  |
| *I addressed the perpetrator later.* |  |  |  |  |  |
| *I changed the subject or shifted the focus of the perpetrator.* |  |  |  |  |  |
| *I reported the perpetrator through official channels ([INSTITUTIONAL REPORTING SYSTEM], course director, dean).* |  |  |  |  |  |
| *I discussed the situation with a trusted advisor (faculty, resident, mentor).* |  |  |  |  |  |
| *I discussed the situation with friends.* |  |  |  |  |  |

1. In the clinical learning environment, how often have you **witnessed** biases and/or microaggressions **directed at another person**? Please provide your best estimate.
   1. Never
   2. A few times
   3. Monthly
   4. Weekly
   5. Daily
   6. Other (please describe):
2. If you have **witnessed** biases and/or microaggressions, did you respond to the situation?
3. Yes
4. No
5. If you did not respond, why? (select all that apply)
6. The hierarchy and power dynamics made me feel that I should not respond.
7. I didn’t know if the victim wanted anyone to step in and respond
8. I couldn’t tell if the victim was bothered or upset by the situation
9. I was worried about grade or other academic consequences
10. I was worried about personal consequences
11. The instance didn’t seem important enough
12. I did not want the perpetrator to get in trouble
13. I knew the perpetrator, and I did not think this behavior was in-character
14. I was not sure if I interpreted the situation appropriately
15. Other:
16. If you did respond, how did you address the individual(s) involved? Please place a check or ‘x’ in the box that best characterizes your views on each statement.

|  | Always | Often | Sometimes | Rarely | Never |
| --- | --- | --- | --- | --- | --- |
| *I displayed discomfort or concern.* |  |  |  |  |  |
| *I addressed the perpetrator immediately.* |  |  |  |  |  |
| *I addressed the perpetrator later.* |  |  |  |  |  |
| *I discussed the situation with the victim.* |  |  |  |  |  |
| *I changed the subject or shifted the focus of the perpetrator.* |  |  |  |  |  |
| *I reported the perpetrator through official channels ([INSTITUTIONAL REPORTING SYSTEM], course director, dean).* |  |  |  |  |  |
| *I discussed the situation with a trusted advisor (faculty, resident, mentor).* |  |  |  |  |  |
| *I discussed the situation with friends.* |  |  |  |  |  |

1. On a scale from 1-5, please rate your confidence in addressing a perpetrator of biases and/or microaggressions **directed at you** after the incident, with 1 being not confident and 5 being extremely confident?
2. 1 – not confident
3. 2 – minimally confident
4. 3 – moderately confident
5. 4 – very confident
6. 5 – extremely confident
7. On a scale from 1-5, please rate your confidence in addressing a perpetrator of biases and/or microaggressions **directed at another person** after the incident, with 1 being not confident and 5 being extremely confident?
8. 1 – not confident
9. 2 – minimally confident
10. 3 – moderately confident
11. 4 – very confident
12. 5 – extremely confident
13. Please rate your knowledge:

| Please rate your knowledge regarding: | Pre-event | | | | |
| --- | --- | --- | --- | --- | --- |
|  | None ---------------------------------- Extensive | | | | |
| Definition and understanding of bias | 1 | 2 | 3 | 4 | 5 |
| Definition and understanding of microaggressions | 1 | 2 | 3 | 4 | 5 |
| Recognition of microaggressions | 1 | 2 | 3 | 4 | 5 |
| Framework for responding to biases and/or microaggressions | 1 | 2 | 3 | 4 | 5 |

1. What is your age?
2. I identify my gender as
3. Female
4. Male
5. Transgender female
6. Transgender male
7. Gender Variant Non-conforming
8. Not listed _______________
9. Prefer not to respond
10. I identify my ethnicity as (select all that apply)
11. American Indian / Alaskan Native
12. Asian
13. Black or African American
14. Hispanic, Latinx or Spanish
15. Middle Eastern / Northern African
16. Pacific islander / Native Hawaiian
17. White
18. Prefer not to answer
19. ________________
20. What clerkships have you completed (please select all that apply):
21. Internal Medicine
22. Obstetrics and Gynecology
23. Surgery
24. Pediatrics
25. Psychiatry
26. Neurology

**POST-WORKSHOP SURVEY**

After completing this workshop, which described the framework of responding to bias and microaggressions using the 5 D’s mnemonic (*display discomfort,* *direct response, delay, delegate, and distraction)*, please indicate your responses to the questions below.

1. Having completed the session on bystander training, on a scale from 1-5, please rate your confidence in addressing a perpetrator of biases and/or microaggressions **directed at you** after the incident, with 1 being not confident and 5 being extremely confident?
   1. 1 – not confident
   2. 2 – minimally confident
   3. 3 – moderately confident
   4. 4 – very confident
   5. 5 – extremely confident
2. Having completed the session on bystander training, on a scale from 1-5, please rate your confidence in addressing a perpetrator of biases and/or microaggressions **directed at another person** after the incident, with 1 being no confident and 5 being extremely confident?
   1. 1 – not confident
   2. 2 – minimally confident
   3. 3 – moderately confident
   4. 4 – very confident
   5. 5 – extremely confident
3. Please rate your knowledge:

| Please rate your knowledge regarding: | Post-event | | | | |
| --- | --- | --- | --- | --- | --- |
|  | None ---------------------------------- Extensive | | | | |
| Definition and understanding of bias | 1 | 2 | 3 | 4 | 5 |
| Definition and understanding of microaggressions | 1 | 2 | 3 | 4 | 5 |
| Recognition of microaggressions | 1 | 2 | 3 | 4 | 5 |
| Framework for responding to biases and/or microaggressions | 1 | 2 | 3 | 4 | 5 |

1. (Select all that apply) How likely are you to respond to biases and/or microaggressions **directed at you** with:

|  | Always | Often | Sometimes | Rarely | Never |
| --- | --- | --- | --- | --- | --- |
| *I will* ***display discomfort*** *or concern.* |  |  |  |  |  |
| *I will address the perpetrator immediately* ***(direct response).*** |  |  |  |  |  |
| *I will address the perpetrator later* ***(delay).*** |  |  |  |  |  |
| *I will change the subject or distract the perpetrator immediately* ***(distract response).*** |  |  |  |  |  |
| *I will report the perpetrator through official channels ([INSTITUTIONAL REPORTING SYSTEM], course director, dean)* ***(delegation response).*** |  |  |  |  |  |
| *I will discuss the situation with a trusted advisor (faculty, resident, mentor)* ***(delegation response).*** |  |  |  |  |  |
| *I will discuss the situation with friends.* |  |  |  |  |  |

1. (Select all that apply) How likely are you to respond to biases and/or microaggressions **directed at another person** with:

|  | Always | Often | Sometimes | Rarely | Never |
| --- | --- | --- | --- | --- | --- |
| *I will* ***display discomfort*** *or concern.* |  |  |  |  |  |
| *I will address the perpetrator immediately* ***(direct response).*** |  |  |  |  |  |
| *I will address the victim immediately* ***(direct response).*** |  |  |  |  |  |
| *I will address the perpetrator or victim* ***later*** ***(direct response).*** |  |  |  |  |  |
| *I will change the subject or distract the perpetrator immediately* ***(distract response).*** |  |  |  |  |  |
| *I will report the perpetrator through official channels ([INSTITUTIONAL REPORTING SYSTEM], course director, dean)* ***(delegation response).*** |  |  |  |  |  |
| *I will discuss the situation with a trusted advisor (faculty, resident, mentor)* ***(delegation response).*** |  |  |  |  |  |
| *I will discuss the situation with friends.* |  |  |  |  |  |

1. What was most helpful about this workshop and why? (free response)
2. How might the speakers make the session more effective in the future? (free response)
3. Please identify one (or a few) response type(s) you are committed to using as a result of this workshop. (free response)
4. The workshop was effective.
   1. Strongly Agree
   2. Agree
   3. Neutral
   4. Disagree
   5. Strongly Disagree

**LONGITUDINAL SURVEY**

For **questions 1-5**, please place a check or ‘x’ in the box that best characterizes your views on each statement.

|  | Strongly Agree | Agree | Neutral | Disagree | Strongly Disagree | I don’t know |
| --- | --- | --- | --- | --- | --- | --- |
| 1. I feel safe in the clinical learning environment at Vanderbilt. |  |  |  |  |  |  |
| 1. Microaggressions are an issue in the clinical learning environment at Vanderbilt that should be addressed. |  |  |  |  |  |  |
| 1. Microaggressions are prevalent in the clinical learning environment at Vanderbilt |  |  |  |  |  |  |
| 1. Bias is an issue in the clinical learning environment at Vanderbilt that should be addressed |  |  |  |  |  |  |
| 1. Bias is prevalent in the clinical learning environment at Vanderbilt. |  |  |  |  |  |  |

1. Over the **past 4-weeks**, how often have you **experienced** biases and/or microaggressions in the clinical learning environment? Please provide your best estimate.
   1. Never
   2. A few times
   3. Monthly
   4. Weekly
   5. Daily
   6. Other (please describe):
2. If you have **experienced** biases and/or microaggressions, did you respond to the situation?
   1. Yes
   2. No
3. If you did not respond, why? (select all that apply)
4. The hierarchy and power dynamics made me feel that I should not respond.
5. I was worried about grade or other academic consequences
6. I was worried about personal consequences
7. The instance didn’t seem important enough
8. I did not want the perpetrator to get in trouble
9. I knew the perpetrator, and I did not think this behavior was in-character
10. I was not sure if I interpreted the situation appropriately
11. Other:
12. If you did respond, how did you address the individual(s) involved? Please place a check or ‘x’ in the box that best characterizes your views on each statement.

|  | Always | Often | Sometimes | Rarely | Never |
| --- | --- | --- | --- | --- | --- |
| *I will* ***display discomfort*** *immediately.* |  |  |  |  |  |
| *I will address the perpetrator immediately* ***(direct response).*** |  |  |  |  |  |
| *I will address the perpetrator later* ***(delayed direct).*** |  |  |  |  |  |
| *I will change the subject or distract the perpetrator immediately* ***(distract response).*** |  |  |  |  |  |
| *I will report the perpetrator through official channels ([INSTITUTIONAL REPORTING SYSTEM], course director, dean)* ***(delegation response).*** |  |  |  |  |  |
| *I will discuss the situation with a trusted advisor (faculty, resident, mentor)* ***(delegation response).*** |  |  |  |  |  |
| *I will discuss the situation with friends.* |  |  |  |  |  |

1. In the **past 4-weeks**, how often have you **witnessed** biases and/or microaggressions in the clinical learning environment? Please provide your best estimate.
   1. Never
   2. A few times
   3. Monthly
   4. Weekly
   5. Daily
   6. Other (please describe):
2. If you have **witnessed** biases and/or microaggressions, did you respond to the situation?
3. Yes
4. No
5. If you did not respond, why? (select all that apply)
6. The hierarchy and power dynamics made me feel that I should not respond.
7. I didn’t know if the victim wanted anyone to step in and respond
8. I couldn’t tell if the victim was bothered or upset by the situation
9. I was worried about grade or other academic consequences
10. I was worried about personal consequences
11. The instance didn’t seem important enough
12. I did not want the perpetrator to get in trouble
13. I knew the perpetrator, and I did not think this behavior was in-character
14. I was not sure if I interpreted the situation appropriately
15. Other:
16. If you did respond, how did you address the individual(s) involved? Please place a check or ‘x’ in the box that best characterizes your views on each statement.

|  | Always | Often | Sometimes | Rarely | Never |
| --- | --- | --- | --- | --- | --- |
| *I will* ***display discomfort*** *or concern.* |  |  |  |  |  |
| *I will address the perpetrator immediately* ***(direct response).*** |  |  |  |  |  |
| *I will address the victim immediately* ***(direct response).*** |  |  |  |  |  |
| *I will address the perpetrator or victim* ***later*** ***(direct response).*** |  |  |  |  |  |
| *I will change the subject or distract the perpetrator immediately* ***(distract response).*** |  |  |  |  |  |
| *I will report the perpetrator through official channels ([INSTITUTIONAL REPORTING SYSTEM], course director, dean)* ***(delegation response).*** |  |  |  |  |  |
| *I will discuss the situation with a trusted advisor (faculty, resident, mentor)* ***(delegation response).*** |  |  |  |  |  |
| *I will discuss the situation with friends.* |  |  |  |  |  |

1. On a scale from 1-5, please rate your confidence in addressing (direct, delegate, distract, or other approach) a perpetrator of biases and/or microaggressions **directed at you** after the incident, with 1 being noy confident and 5 being extremely confident?
2. 1 – not confident
3. 2 – minimally confident
4. 3 – moderately confident
5. 4 – very confident
6. 5 – extremely confident
7. On a scale from 1-5, please rate your confidence in addressing (direct, delegate, distract, or other approach) a perpetrator of biases and/or microaggressions **directed at another person** after the incident, with 1 being not confident and 5 being extremely confident?
8. 1 – not confident
9. 2 – minimally confident
10. 3 – moderately confident
11. 4 – very confident
12. 5 – extremely confident
13. What is your age?
14. I identify my gender as
15. Female
16. Male
17. Transgender female
18. Transgender male
19. Gender Variant Non-conforming
20. Not listed _______________
21. Prefer not to respond
22. I identify my ethnicity as (select all that apply)
23. American Indian / Alaskan Native
24. Asian
25. Black or African American
26. Hispanic, Latinx or Spanish
27. Middle Eastern / Northern African
28. Pacific islander / Native Hawaiian
29. White
30. Multiple ethnicities
31. Prefer not to answer
32. What clerkship were you on in the past month?
    1. Internal Medicine
    2. Obstetrics and Gynecology
    3. Surgery
    4. Pediatrics
    5. Psychiatry
    6. Neurology
33. Do you think more training for medical students on responding to microaggressions would be a helpful addition to the curriculum?
34. Yes
35. Somewhat
36. No, this is sufficient training
37. No, I don’t think this training is necessary
38. I don’t know
39. What component of the workshops was most effective (select up to 3):
    1. Background definitions
    2. The D’s Framework
    3. Case examples
    4. Roleplay
    5. Generating possible responses with peers
    6. Other: _______________
