# Supplementary material for: Becoming Active Bystanders and Advocates: Teaching Medical Students to Respond to Bias in the Clinical Setting
Source: MedEdPORTAL. 2021 Aug 19;17:11175. doi: 10.15766/mep_2374-8265.11175 (PMC8374028; doi:10.15766/mep_2374-8265.11175)
Supplement: Supplementary file 1 — Bystander Training.pptxFacilitator Guide.docxResponse Framework Handout.docxExample Cases.docxSurveys.docxFocus Group Facilitator Guide.docx [file mep_2374-8265.11175-s001.zip › F. Focus Group Facilitator Guide.docx]

**Bystander Training Focus Group Facilitator Guide**

Introduction script:

Welcome to the mentee session.  Thank you for taking the time to join us to discuss the Bystander Training session.  For those of you who do not know me, my name is [insert name] and I am a [insert role and relevant titles or training]. With the help of additional supporting faculty members, we worked together to develop this training session and designed the surveys to assess your attitude towards acting on and responding to instances of witnessed or experienced bias and/or microaggressions in the clinical setting.

We have invited you here today to gather more information on what you liked and didn’t like about the program, and to better understand how it can be improved for future groups. There are no right or wrong answers, as we simply seek to understand your different points of view.  Please feel free to share your perspectives candidly, and please help us in creating a safe space for one another by being respectful of everyone’s views and opinions.

So that we do not miss any of your comments, we would like to record an audio transcript of this discussion.  Very thoughtful discussions usually occur during these sessions and I am not able to write quickly enough to capture all of your thoughts accurately as well as conduct the session. Any identifiers shared during the session will be removed from the transcript, and the qualitative data will therefore remain anonymous. The final report will be used to improve the program going forward.

Before beginning the recording, let’s begin with an introduction of your name as well as what clerkships you have completed thus far.

Open ended questions:

- In reflecting on the training session, what were your initial reactions to what we discussed?
- Based on your experiences, do you think this training session was necessary? Why or why not?
- What do you think worked well and was effective within the training session?
- What could be improved within the training session?
- What were you hoping to get out of the program?
- How do you feel about the timing of the training session in terms of implementing it after your first clerkship of second year?
- How do you feel about a student giving the training? How do you think your experience would have been affected if it were given by a faculty?
- Are there other areas or topics you wished the program addressed? If so, what are they?
- Would you recommend this training to all medical students at Vanderbilt? What about to students in other medical schools?

*Free time for additional points of discussion. At the conclusion of discussion, stop recording.*

Closing remarks: Thank you so much for attending this session. We are very thankful for your participation and contributions to improving this program for future medical students.
